# The Burden of Pancreatic Cancer in Five East Asian Countries From 1990 to 2021 and Its Prediction up to 2036: A Systemic Analysis of the Global Burden of Diseases Study 2021

**DOI:** 10.1002/cam4.70656

**Published:** 2025-12-07

**Authors:** Tianhao Guo, Wenjian Zhu, Yifan Hui, Yuhan Wang, Tingting Zhou, Weixing Shen, Liu Li, Yu Yang, Haibo Cheng

**Affiliations:** ^1^ Institute of Health and Regimen Jiangsu Open University Nanjing Jiangsu People's Republic of China; ^2^ Jiangsu Collaborative Innovation Center of Traditional Chinese Medicine Prevention and Treatment of Tumor Nanjing Jiangsu People's Republic of China; ^3^ The First Clinical Medical College Nanjing University of Chinese Medicine Nanjing Jiangsu People's Republic of China; ^4^ Wangjing Hospital of China Academy of Chinese Medical Sciences Beijing People's Republic of China; ^5^ Department of Oncology Affiliated Hospital of Nanjing University of Chinese Medicine Nanjing Jiangsu People's Republic of China

**Keywords:** age‐period‐cohort analysis, disease burden, epidemiological study, Global Burden of Disease Study, joinpoint regression, pancreatic cancer

## Abstract

**Background:**

Pancreatic cancer (PC) presents a significant challenge for prevention and treatment, posing a serious threat to the health and lives of patients. The five East Asian countries (China, Japan, North Korea, South Korea, and Mongolia) represent one of the most significant regions globally in terms of PC burden.

**Methods:**

We retrieved data from the Global Burden of Disease (GBD) Study 2021 regarding the prevalence, incidence, mortality, years lived with disability (YLDs), years of life lost (YLLs), and disability‐adjusted life years (DALYs) associated with PC in these five East Asian countries from 1990 to 2021. We employed joinpoint, age‐period‐cohort (APC), and decomposition analysis methods to assess the epidemiological characteristics of PC. To project the future burden of PC through 2036, we applied two prediction models: Autoregressive Integrated Moving Average (ARIMA) and Bayesian age‐period‐cohort (BAPC) models.

**Results:**

China recorded the highest incidence, prevalence, mortality, YLLs, YLDs, and DALYs among the five East Asian countries in both 1990 and 2021. Japan exhibited the highest age‐standardized incidence rate (ASIR), age‐standardized mortality rate (ASMR), age‐standardized prevalence rate (ASPR), and age‐standardized YLDs rate in both 1990 and 2021. Mongolia experienced significant increases in the number and rates of incidence, prevalence, death, YLLs, YLDs, and DALYs from 1990 to 2021. The age group with the highest prevalence, incidence, mortality, YLDs, YLLs, and DALYs rates across the five East Asian countries was consistently those aged 70 and older. The incidence rate across the five countries is influenced by aging populations, surpassing global averages. Projections for 2030 and 2036 suggest that Japan will have the highest ASPR (13.23 in 2030 and 13.85 in 2036), ASIR (12.14 in 2030 and 12.53 in 2036), and ASMR (10.97 in 2030 and 11.39 in 2036) among the countries.

**Conclusion:**

The disease burden of PC in the five East Asian countries has steadily increased over the past three decades, particularly among older adults due to population aging.

## 1 Introduction

Pancreatic cancer (PC) accounted for 2.6% of all cancer diagnoses worldwide in 2022, ranking as the twelfth most frequently diagnosed cancer [1]. It is the sixth leading cause of cancer‐related death, contributing to 4.8% of global cancer mortality [1]. The incidence of PC has been rising annually [2], particularly in Asian countries [3, 4].

The five East Asian countries (China, Japan, North Korea, South Korea, and Mongolia) collectively represent approximately 22% of the global population [5]. These countries share similar geographical locations, cultural influences, dietary habits, and lifestyle patterns.

PC presents significant challenges for prevention and treatment, posing a serious threat to the life and health of patients [6]. However, the availability of PC registries remains inadequate, and the epidemiological reports from the five East Asian countries are limited. The lack of epidemiological data complicates the formulation of effective public health policies and cancer prevention strategies.

The 2021 Global Burden of Diseases, Injuries, and Risk Factors Study (GBD) provided a cause‐of‐death analysis that estimated mortality rates and years of life lost (YLLs) for 288 causes of death [5]. This analysis included data stratified by age, gender, and location across 204 countries and regions, as well as 811 subnational areas, from 1990 to 2021 [5, 7].

This study comprehensively examines the disease burden of PC in the five East Asian countries from 1990 to 2021, analyzing prevalence, incidence, mortality, years of life lived with disability (YLDs), YLLs, and disability‐adjusted life years (DALYs). Additionally, we employ the Autoregressive Integrated Moving Average (ARIMA) and Bayesian age‐period‐cohort (BAPC) models to predict disease trends through 2036. This study aims to provide decision‐makers with valuable insights to assess the overall burden of PC in the five East Asian countries, enabling the development of targeted prevention strategies and the equitable allocation of public health resources.

## 2 Methods

### 2.1 Data Source

The data for this study were obtained from the Global Health Data Exchange GBD Results Tool (https://vizhub.healthdata.org/gbd‐results/) for the years 1990 to 2021. The GBD platform provides access to all age‐standardized rates and age‐specific rates, including data with 95% uncertainty intervals (UI).

### 2.2 Definition

PC was defined as all cancers classified under C25, according to the 10th revision of the International Classification of Diseases (ICD) (https://www.cdc.gov/nchs/icd/icd‐10‐cm.htm).

### 2.3 Data Analysis

The statistical methods used for GBD 2021 have been extensively detailed in prior literature [5]. Trends in prevalence, incidence, mortality, YLDs, YLLs, and DALYs from 1990 to 2021 were analyzed by year, gender, and age group.

The average annual percent change (AAPC) and annual percentage change (APC), along with their respective 95% confidence intervals (CIs), were calculated to assess trends in the age‐standardized incidence rate (ASIR), age‐standardized mortality rate (ASMR), and age‐standardized disability‐adjusted life year rate (ASDR) using Joinpoint regression analysis (Joinpoint Regression Program, Version 5.1.0). Age‐standardized rates (ASRs) were computed using the GBD world population age standard.

The decomposition method was applied to partition the changes in the total number of deaths into contributions from population growth, population aging, and changes in mortality. Detailed descriptions of this approach are available in published literatures [8, 9, 10, 11, 12, 13].

The ARIMA model, comprising both Autoregressive (AR) and Moving Average (MA) components, was utilized [14]. This time‐series model assumes that data are time‐dependent random variables whose autocorrelation can be modeled using ARIMA. Predictions were made based on past data trends, as described in prior studies [15].

To project the burden of PC through 2036, the BAPC model was employed using the BAPC and INLA packages in R. Further details on this method are documented in previous studies [16]. Data processing and analysis were performed using R (version 4.4.1) along with Zstats v1.0 (www.zstats.net), with statistical significance set at a two‐tailed *p*‐value < 0.05 [17].

### 2.4 Role of the Funding Source

The funders of this study had no role in study design, data collection, data analysis, data interpretation, or report writing. All authors had full access to all the study data, and the corresponding author assumed responsibility for the decision to submit the manuscript for publication.

## 3 Results

### 3.1 Overall Burden

In China, ASIR increased from 4.54 (95% UI: 3.84–5.29) per 100,000 population in 1990 to 5.64 (95% UI: 4.52–6.84) in 2021. In Japan, ASIR rose from 9.52 (95% UI: 8.92–9.86) in 1990 to 11.55 (95% UI: 10.13–12.34) in 2021. In South Korea, ASIR showed minimal change, from 8.22 (95% UI: 6.98–9.52) in 1990 to 8.23 (95% UI: 6.45–9.99) in 2021. In North Korea, the ASIR slightly increased from 3.36 (95% UI: 2.44–4.56) in 1990 to 3.47 (95% UI: 2.22–4.67) in 2021. In Mongolia, the ASIR rose significantly from 1.41 (95% UI: 1.07–1.86) in 1990 to 7.40 (95% UI: 5.46–9.86) in 2021 (Table 1). Japan had the highest ASIR in 1990 and 2021. In 1990 and 2021, China's ASIR was lower than the global ASIR but higher than the ASIR of Asia. South Korea's ASIR was consistently higher than the global ASIR and the ASIR of Asia in both 1990 and 2021. Conversely, North Korea had ASIRs both below the global ASIR and the ASIR of Asia in 1990 and 2021. Mongolia's ASIR was below the global ASIR and the ASIR of Asia in 1990 but surpassed them by 2021.

**TABLE 1:** Incidence of pancreatic cancer between 1990 and 2021 at the global, regional, and five East‐Asian countries levels.

Location	1990 incident cases (95% UI)			1990 age‐standardized rates per 100,000 people (95% UI)			2021 incident cases (95% UI)			2021 age‐standardized rates per 100,000 people (95% UI)		
	Total	Male	Female	Total	Male	Female	Total	Male	Female	Total	Male	Female
Global	207,905 (196,649, 217,778)	110,396 (104,542, 116,510)	97,510 (90,498, 103,233)	5.47 (5.16, 5.73)	6.3 (5.95, 6.62)	4.71 (4.34, 4.99)	508,533 (462,091, 547,208)	273,617 (250,808, 299,348)	234,916 (205,149, 255,435)	5.96 (5.39, 6.42)	6.96 (6.37, 7.59)	5.05 (4.41, 5.49)
SDI												
High SDI	97,522 (92,266, 100,694)	48,924 (47,493, 50,237)	48,598 (44,692, 50,831)	8.75 (8.29, 9.03)	10.55 (10.2, 10.83)	7.31 (6.77, 7.63)	217,146 (193,741, 231,693)	110,810 (103,440, 116,614)	106,336 (90,284, 115,699)	10 (9.08, 10.61)	11.54 (10.8, 12.13)	8.58 (7.5, 9.23)
High‐middle SDI	66,491 (62,906, 70,238)	36,647 (34,070, 39,388)	29,844 (27,832, 32,031)	6.75 (6.38, 7.14)	8.52 (7.95, 9.15)	5.33 (4.94, 5.72)	148,297 (132,454, 164,406)	82,216 (72,058, 94,434)	66,081 (57,507, 74,468)	7.47 (6.67, 8.27)	9.24 (8.12, 10.55)	5.93 (5.18, 6.69)
Middle SDI	32,383 (29,495, 35,580)	18,276 (16,223, 20,590)	14,108 (12,451, 15,847)	3.18 (2.92, 3.48)	3.63 (3.26, 4.05)	2.75 (2.44, 3.08)	103,576 (91,436, 116,663)	59,273 (51,107, 68,783)	44,303 (38,490, 50,373)	3.88 (3.43, 4.36)	4.67 (4.04, 5.4)	3.16 (2.74, 3.59)
Low‐middle SDI	8316 (7055, 9706)	4757 (3956, 5547)	3559 (2919, 4247)	1.39 (1.18, 1.62)	1.54 (1.29, 1.79)	1.23 (1.01, 1.46)	31,148 (28,919, 33,651)	16,924 (15,572, 18,316)	14,224 (12,912, 15,451)	2.2 (2.04, 2.37)	2.46 (2.27, 2.66)	1.95 (1.76, 2.11)
Low SDI	2902 (2251, 3479)	1634 (1245, 1976)	1268 (943, 1584)	1.31 (1.02, 1.57)	1.43 (1.1, 1.73)	1.18 (0.87, 1.46)	7830 (6494, 9394)	4113 (3374, 5078)	3717 (2999, 4455)	1.59 (1.33, 1.9)	1.68 (1.39, 2.05)	1.51 (1.21, 1.81)
Asia	73,435 (66,358, 81,421)	42,598 (37,694, 47,885)	30,837 (26,958, 35,525)	3.81 (3.45, 4.2)	4.44 (3.97, 4.96)	3.19 (2.8, 3.64)	234,559 (205,778, 264,948)	134,219 (115,495, 156,089)	100,340 (83,018, 115,707)	4.78 (4.19, 5.39)	5.77 (5, 6.66)	3.87 (3.19, 4.45)
China	37,818 (31,791, 44,068)	22,555 (18,204, 27,474)	15,262 (12,235, 18,866)	4.54 (3.84, 5.29)	5.55 (4.55, 6.64)	3.64 (2.93, 4.48)	118,665 (94,623, 144,663)	72,280 (54,334, 92,975)	46,386 (34,923, 59,339)	5.64 (4.52, 6.84)	7.29 (5.55, 9.24)	4.18 (3.15, 5.34)
Japan	16,075 (15,142, 16,620)	8834 (8513, 9075)	7240 (6509, 7670)	9.52 (8.92, 9.86)	12.26 (11.77, 12.61)	7.4 (6.65, 7.84)	46,502 (39,057, 50,709)	22,860 (20,921, 24,090)	23,643 (17,883, 26,933)	11.55 (10.13, 12.34)	13.7 (12.67, 14.35)	9.57 (7.82, 10.54)
South Korea	2371 (2016, 2728)	1372 (1135, 1629)	999 (826, 1168)	8.22 (6.98, 9.52)	11.38 (9.5, 13.55)	6.11 (5.05, 7.19)	7784 (6114, 9429)	4170 (3301, 5134)	3614 (2687, 4441)	8.23 (6.45, 9.99)	10.15 (8, 12.53)	6.61 (4.97, 8.1)
North Korea	553 (393, 758)	293 (205, 413)	259 (184, 367)	3.36 (2.44, 4.56)	4.38 (3.13, 6.01)	2.68 (1.91, 3.7)	1165 (747, 1573)	662 (453, 917)	502 (280, 729)	3.47 (2.22, 4.67)	4.56 (3.11, 6.17)	2.61 (1.45, 3.76)
Mongolia	15 (11, 19)	8 (6, 11)	7 (5, 9)	1.41 (1.07, 1.86)	1.63 (1.23, 2.16)	1.2 (0.87, 1.6)	172 (128, 228)	100 (73, 132)	73 (53, 96)	7.4 (5.46, 9.86)	9.54 (6.99, 12.59)	5.76 (4.2, 7.67)

In China, the ASMR increased from 4.83 (95% UI: 4.10–5.61) per 100,000 population in 1990 to 5.72 (95% UI: 4.59–6.91) in 2021. Similarly, Japan's ASMR rose from 8.96 (95% UI: 8.39–9.28) in 1990 to 10.28 (95% UI: 9.08–10.97) in 2021. South Korea's ASMR decreased from 8.53 (95% UI: 7.26–9.94) in 1990 to 7.51 (95% UI: 5.88–9.14) in 2021. North Korea's ASMR remained relatively stable, increasing slightly from 3.54 (95% UI: 2.60–4.80) in 1990 to 3.58 (95% UI: 2.27–4.80) in 2021. Mongolia experienced a significant increase in ASMR, rising from 1.50 (95% UI: 1.14–1.97) in 1990 to 7.88 (95% UI: 5.86–10.51) in 2021 (Table 2). Among the five East Asian countries, Japan had the highest ASMR in both 1990 and 2021. China's ASMR remained below the global ASMR in both 1990 and 2021, whereas South Korea's ASMR consistently exceeded it. North Korea's ASMR was below the global ASMR in 1990 and 2021, whereas Mongolia's ASMR surpassed it in 2021 after being below it in 1990.

**TABLE 2:** Deaths of pancreatic cancer between 1990 and 2021 at the global, regional, and five East‐Asian countries levels.

Location	1990 deaths cases (95% UI)			1990 age‐standardized rates per 100,000 people (95% UI)			2021 deaths cases (95% UI)			2021 age‐standardized rates per 100,000 people (95% UI)		
	Total	Male	Female	Total	Male	Female	Total	Male	Female	Total	Male	Female
Global	211,613 (199,990, 221,951)	111,023 (105,147, 117,267)	100,590 (93,631, 106,565)	5.66 (5.33, 5.93)	6.48 (6.13, 6.82)	4.9 (4.52, 5.19)	505,752 (461,224, 543,899)	270,037 (247,470, 295,173)	235,715 (206,199, 256,637)	5.95 (5.4, 6.41)	6.93 (6.35, 7.55)	5.06 (4.43, 5.51)
SDI												
High SDI	97,179 (91,896, 100,353)	48,121 (46,705, 49,419)	49,058 (45,081, 51,302)	8.7 (8.22, 8.98)	10.47 (10.12, 10.75)	7.3 (6.75, 7.62)	206,593 (184,626, 220,562)	104,897 (97,804, 110,528)	101,696 (86,453, 110,700)	9.37 (8.52, 9.96)	10.85 (10.15, 11.43)	8.03 (7.03, 8.65)
High‐middle SDI	68,843 (65,128, 72,746)	37,385 (34,751, 40,201)	31,458 (29,352, 33,734)	7.09 (6.69, 7.5)	8.93 (8.33, 9.58)	5.65 (5.24, 6.07)	151,080 (135,113, 166,837)	82,661 (72,735, 94,545)	68,419 (59,511, 76,997)	7.61 (6.8, 8.4)	9.37 (8.27, 10.67)	6.1 (5.33, 6.87)
Middle SDI	33,584 (30,683, 36,872)	18,732 (16,676, 21,095)	14,851 (13,148, 16,671)	3.42 (3.14, 3.72)	3.87 (3.49, 4.31)	2.98 (2.65, 3.33)	106,766 (94,342, 119,906)	60,404 (52,205, 69,895)	46,363 (40,175, 52,676)	4.06 (3.6, 4.54)	4.86 (4.2, 5.6)	3.34 (2.89, 3.79)
Low‐middle SDI	8671 (7355, 10,121)	4924 (4102, 5738)	3748 (3069, 4459)	1.5 (1.27, 1.74)	1.64 (1.37, 1.9)	1.34 (1.1, 1.59)	32,553 (30,235, 35,163)	17,514 (16,116, 18,930)	15,039 (13,636, 16,346)	2.35 (2.17, 2.53)	2.61 (2.42, 2.82)	2.1 (1.9, 2.28)
Low SDI	3029 (2351, 3634)	1698 (1301, 2054)	1331 (986, 1658)	1.41 (1.1, 1.7)	1.54 (1.2, 1.86)	1.28 (0.94, 1.58)	8199 (6800, 9862)	4272 (3512, 5266)	3926 (3163, 4728)	1.73 (1.45, 2.07)	1.81 (1.5, 2.21)	1.64 (1.32, 1.98)
Asia	74,009 (66,873, 82,214)	42,607 (37,636, 47,982)	31,402 (27,490, 36,104)	3.92 (3.54, 4.33)	4.56 (4.07, 5.1)	3.3 (2.9, 3.76)	231,770 (203,197, 261,662)	132,087 (113,648, 153,759)	99,683 (83,000, 115,444)	4.75 (4.17, 5.34)	5.74 (4.98, 6.63)	3.85 (3.18, 4.45)
China	38,883 (32,790, 45,260)	22,937 (18,492, 27,879)	15,946 (12,876, 19,707)	4.83 (4.1, 5.61)	5.91 (4.88, 7.06)	3.9 (3.15, 4.81)	119,602 (95,654, 145,218)	72,159 (54,384, 92,255)	47,443 (35,778, 60,492)	5.72 (4.59, 6.91)	7.37 (5.64, 9.3)	4.29 (3.23, 5.46)
Japan	15,084 (14,196, 15,595)	8261 (7970, 8451)	6823 (6177, 7196)	8.96 (8.39, 9.28)	11.56 (11.07, 11.85)	6.97 (6.3, 7.36)	42,065 (35,527, 45,867)	20,709 (18,971, 21,658)	21,356 (16,246, 24,283)	10.28 (9.08, 10.97)	12.3 (11.43, 12.8)	8.45 (6.96, 9.31)
South Korea	2390 (2043, 2752)	1369 (1141, 1624)	1020 (847, 1191)	8.53 (7.26, 9.94)	11.87 (9.95, 14.13)	6.37 (5.29, 7.48)	7101 (5596, 8633)	3817 (3007, 4690)	3284 (2442, 4050)	7.51 (5.88, 9.14)	9.35 (7.35, 11.52)	5.99 (4.48, 7.36)
North Korea	565 (406, 773)	294 (207, 411)	271 (194, 379)	3.54 (2.6, 4.8)	4.61 (3.32, 6.31)	2.85 (2.05, 3.93)	1187 (756, 1601)	663 (453, 918)	524 (289, 763)	3.58 (2.27, 4.8)	4.7 (3.2, 6.32)	2.71 (1.49, 3.95)
Mongolia	16 (12, 20)	8 (6, 11)	7 (5, 10)	1.5 (1.14, 1.97)	1.71 (1.31, 2.25)	1.3 (0.93, 1.74)	177 (132, 236)	101 (75, 134)	76 (56, 100)	7.88 (5.86, 10.51)	10.08 (7.48, 13.24)	6.22 (4.58, 8.27)

China's age‐standardized prevalence rate (ASPR) increased from 3.55 (95% UI: 2.99–4.14) per 100,000 population in 1990 to 4.53 (95% UI: 3.60–5.50) in 2021. Japan's ASPR increased from 8.99 (95% UI: 8.47–9.38) in 1990 to 12.28 (95% UI: 10.64–13.21) in 2021. In South Korea, the ASPR increased from 6.41 (95% UI: 5.44–7.41) in 1990 to 8.02 (95% UI: 6.36–9.78) in 2021. North Korea's ASPR increased from 2.70 (95% UI: 1.93–3.71) in 1990 to 2.91 (95% UI: 1.86–3.93) in 2021. Mongolia exhibited the most pronounced increase, with its ASPR growing from 1.07 (95% UI: 0.81–1.40) in 1990 to 5.72 (95% UI: 4.27–7.58) in 2021 (Table S1). Among the five countries, Japan consistently had the highest ASPR in 1990 and 2021. China's ASPR remained below the global ASPR in both 1990 and 2021, whereas South Korea's ASPR exceeded it. North Korea's ASPR was below the global ASPR in 1990 and 2021. Mongolia's ASPR surpassed the global ASPR by 2021 after being below it in 1990.

China's age‐standardized YLDs rate increased from 0.95 (95% UI: 0.63–1.30) per 100,000 population in 1990 to 1.17 (95% UI: 0.75–1.61) in 2021. Japan's age‐standardized YLDs rate increased from 1.94 (95% UI: 1.38–2.53) in 1990 to 2.39 (95% UI: 1.68–3.16) in 2021. In South Korea, the age‐standardized YLDs rate increased marginally from 1.63 (95% UI: 1.11–2.24) in 1990 to 1.68 (95% UI: 1.09–2.41) in 2021. North Korea experienced a slight increase from 0.72 (95% UI: 0.46–1.04) in 1990 to 0.75 (95% UI: 0.46–1.13) in 2021. Mongolia recorded a sharp rise, from 0.30 (95% UI: 0.19–0.44) in 1990 to 1.51 (95% UI: 0.94–2.17) in 2021 (Table S2). Japan maintained the highest age‐standardized YLDs rate in both years. The age‐standardized YLDs rate of China was below the global rate in both 1990 and 2021, whereas South Korea exceeded it. The age‐standardized YLDs rate of North Korea was below the global rate in both 1990 and 2021. Mongolia's age‐standardized YLDs rate surpassed the global rate by 2021 after being below it in 1990.

China's age‐standardized YLLs rate increased from 122.20 (95% UI: 102.85–142.16) per 100,000 population in 1990 to 136.06 (95% UI: 107.36–165.26) in 2021. Japan's rate rose from 195.60 (95% UI: 186.65–200.81) in 1990 to 212.92 (95% UI: 194.11–223.14) in 2021. South Korea's rate decreased from 202.54 (95% UI: 173.28–233.18) in 1990 to 153.74 (95% UI: 123.11–186.55) in 2021. North Korea showed a slight increase from 92.90 (95% UI: 66.01–127.89) in 1990 to 96.07 (95% UI: 61.29–131.55) in 2021. Mongolia's rate increased dramatically from 38.31 (95% UI: 29.11–50.32) in 1990 to 197.47 (95% UI: 148.26–261.87) in 2021 (Table S3). South Korea had the highest age‐standardized YLL rate among the five East Asian countries in 1990; however, Japan held this distinction in 2021. In 1990, the age‐standardized YLLs rate of China and Mongolia was initially below the global rate but surpassed the global rate by 2021, and that of North Korea remained below the global rate in 1990 and 2021.

In China, the ASDR increased from 123.16 (95% UI: 103.69–143.27) per 100,000 population in 1990 to 137.23 (95% UI: 108.15–166.74) in 2021. Japan's ASDR rose from 197.54 (95% UI: 188.57–202.98) in 1990 to 215.31 (95% UI: 196.04–225.71) in 2021. Conversely, in South Korea, the ASDR decreased from 204.18 (95% UI: 174.62–234.83) in 1990 to 155.42 (95% UI: 124.26–188.67) in 2021. In North Korea, the ASDR increased from 93.62 (95% UI: 66.55–128.98) in 1990 to 96.83 (95% UI: 61.86–132.78) in 2021. Similarly, in Mongolia, the ASDR rose significantly from 38.61 (95% UI: 29.32–50.70) in 1990 to 198.98 (95% UI: 149.16–263.83) in 2021 (Table S4). In 1990, South Korea recorded the highest ASDR among the five East Asian countries, while in 2021, Japan reported the highest ASDR. The ASDRs of China and Mongolia were below the global ASDR in 1990 but exceeded the global ASDR by 2021. In contrast, the ASDR of North Korea remained below the global ASDR in both 1990 and 2021.

### 3.2 Descriptive Analysis From Age Perspective

**FIGURE 1:**
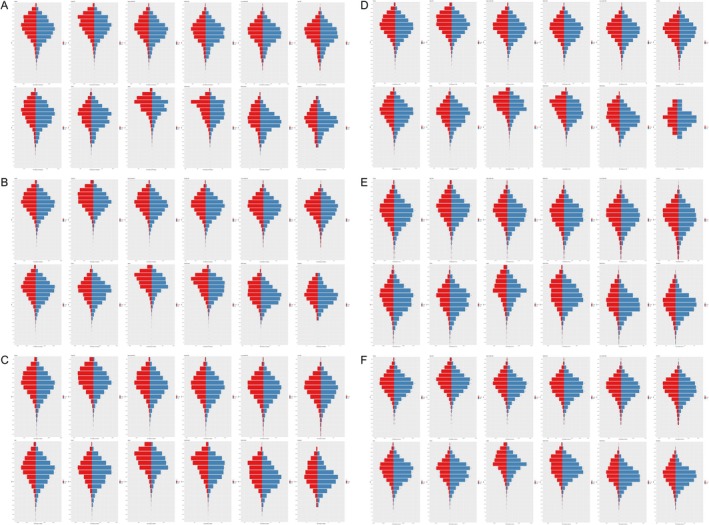
Age‐specific numbers in the five East Asian countries. (A) Age‐specific prevalence, (B) age‐specific incidence, (C) age‐specific mortality, (D) age‐specific years lived with disability (YLDs), (E) age‐specific years of life lost (YLLs), (F) age‐specific disability‐adjusted life years (DALYs).

In 2021, the age group with the highest prevalence of PC in China for females and males was 65–69. In Japan, the age group with the highest prevalence for both genders was 80–84, while in South Korea, the highest prevalence was also observed in the 80–84 age group of both females and males. In North Korea, the highest prevalence age group for females was 60–64, whereas for males, it was 55–59. In Mongolia, the age group with the highest prevalence for females and males was 60–64, with an additional peak for males in the 55–59 age group (Figure 1A).

In 2021, the highest incidence age group for females in China was 70–74, while for males, it was 65–69. In Japan, the age group with the highest incidence for females was 85–89, and for males, it was 70–74. In South Korea, females exhibited the highest incidence in the 80–84 age group, while males showed the highest incidence in the 75–79 age group. In North Korea, both females and males had the highest incidence in the 60–64 age group. Similarly, in Mongolia, the age group with the highest incidence for both females and males was 60–64 (Figure 1B).

In 2021, the age group with the highest mortality in China for females was 70–74, and for males, it was 65–69. In Japan, the age group with the highest mortality for females was 85–89, whereas for males, it was 70–74. In South Korea, the age group with the highest mortality for females was 80–84, whereas for males, it was 75–79. In North Korea, females had the highest mortality in the 75–79 age group, while males had the highest mortality in the 60–64 age group. In Mongolia, the age group with the highest mortality for both females and males was 60–64 (Figure 1C).

In 2021, the age group with the highest YLDs in China for both females and males was 65–69. In Japan, the age group with the highest YLDs for females was 85–89, while for males, it was 70–74. In South Korea, females experienced the highest YLDs in the 80–84 age group, whereas males experienced the highest YLDs in the 65–69 age group. In North Korea, the age group with the highest YLDs for both females and males was 60–64. Similarly, in Mongolia, the highest YLDs for both genders were observed in the 60–64 age group (Figure 1D).

In 2021, the age group with the highest YLLs in China for both females and males in China was 65–69. In Japan, the highest YLLs were observed in the 70–74 age group for both genders. In South Korea, the age group with the highest YLLs for females was 75–79, while for males, it was 60–64. In North Korea, the age group with the highest YLLs for females was 60–64, and for males, it was 50–54. In Mongolia, the age group with the highest YLLs for females was 60–64, whereas for males, it was 55–59 (Figure 1E).

In 2021, the age group with the highest DALYs in China for both females and males was 65–69. In Japan, the highest DALYs were observed in the 70–74 age group for both genders. In South Korea, the age group with the highest DALYs for females was 75–79, whereas for males, it was 60–64. In North Korea, the age group with the highest DALYs for females was 60–64, and for males, it was 50–54. In Mongolia, the age group with the highest DALYs for females was 60–64, whereas for males, it was 55–59 (Figure 1F).

**FIGURE 2:**
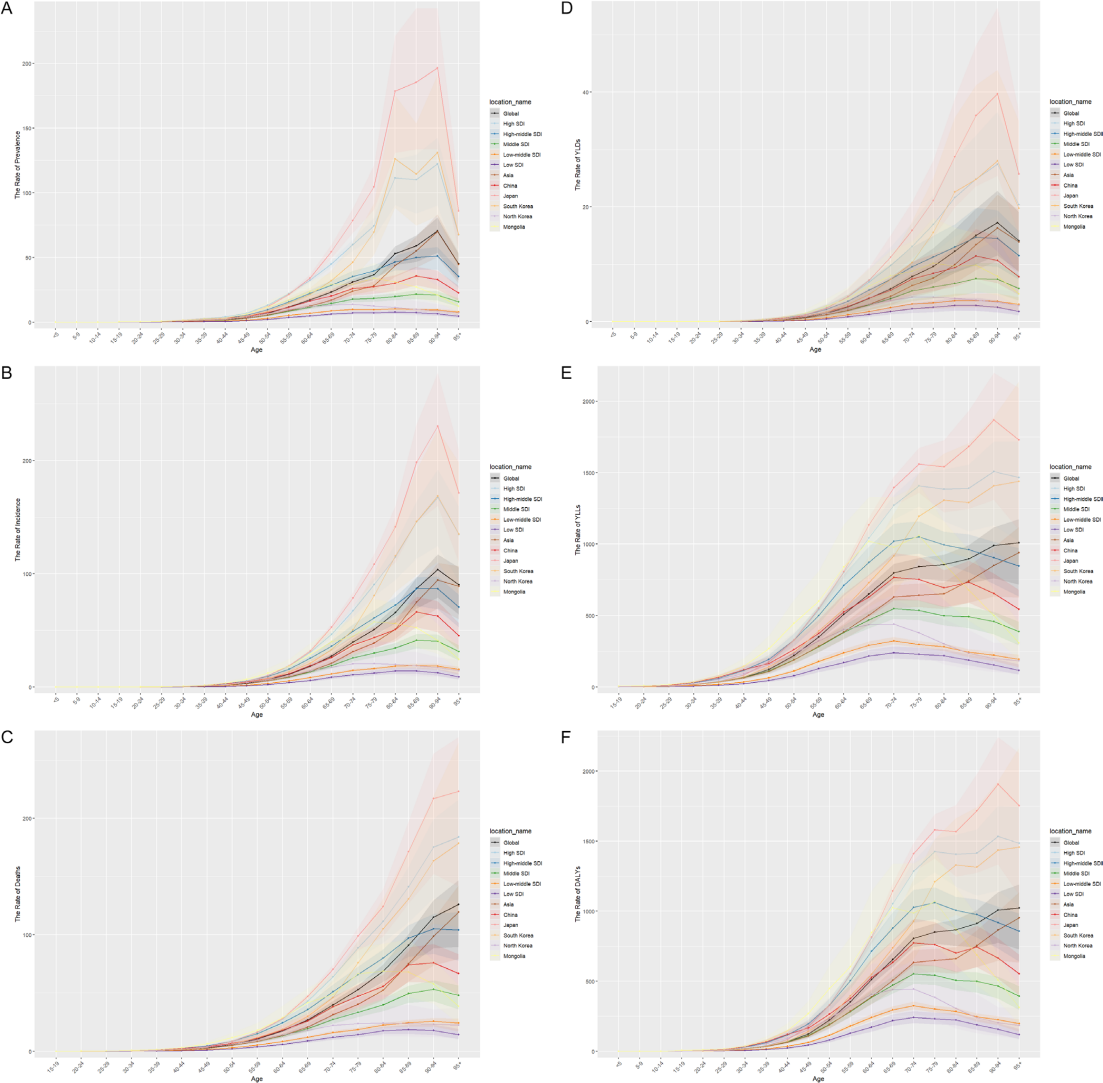
Age‐specific rates in the five East Asian countries. (A) Age‐specific prevalence rate, (B) age‐specific incidence rate, (C) age‐specific death rate, (d) age‐specific YLDs, (E) age‐specific YLLs, (F) age‐specific DALYs.

In 2021, the age group with the highest prevalence rate in China was 85–89. In Japan and South Korea, the highest prevalence rate was observed in the 90–94 age group. North Korea had the highest prevalence rate in the 70–74 age group, while Mongolia reported the highest prevalence rate in the 75–79 age group (Figure 2A).

In 2021, the age group with the highest incidence rate in China was 85–89. In Japan and South Korea, the highest incidence rate occurred in the 90–94 age group. For North Korea and Mongolia, the age group with the highest incidence rate was 75–79 (Figure 2B).

In 2021, the age group with the highest mortality rate in China was 90–94. In Japan and South Korea, the highest mortality rate was observed in individuals aged 95 years or older. For Mongolia and North Korea, the age group with the highest mortality rate was 80–84 (Figure 2C).

In 2021, the age group with the highest YLDs rate in China was 85–89. In Japan and South Korea, the highest YLDs rate was observed in the 90–94 age group. North Korea reported the highest YLDs rate in the 70–74 age group, while in Mongolia, it was 75–79 (Figure 2D).

The age group with the highest YLLs rate in China and North Korea in 2021 was 70–74. In Japan, the highest YLLs rate was observed in the 90–94 age group, whereas in South Korea, it was observed in individuals aged 95 years or older. Mongolia reported the highest YLLs rate in the 75–79 age group (Figure 2E).

In 2021, the age group with the highest DALYs rate in China and North Korea was 70–74. In Japan, the highest DALYs rate was observed in the 90–94 age group, whereas in South Korea, it was observed in individuals aged 95 years or older. In Mongolia, the age group with the highest DALYs rate was 75–79 (Figure 2F).

### 3.3 Descriptive Analysis From a Period Perspective

**FIGURE 3:**
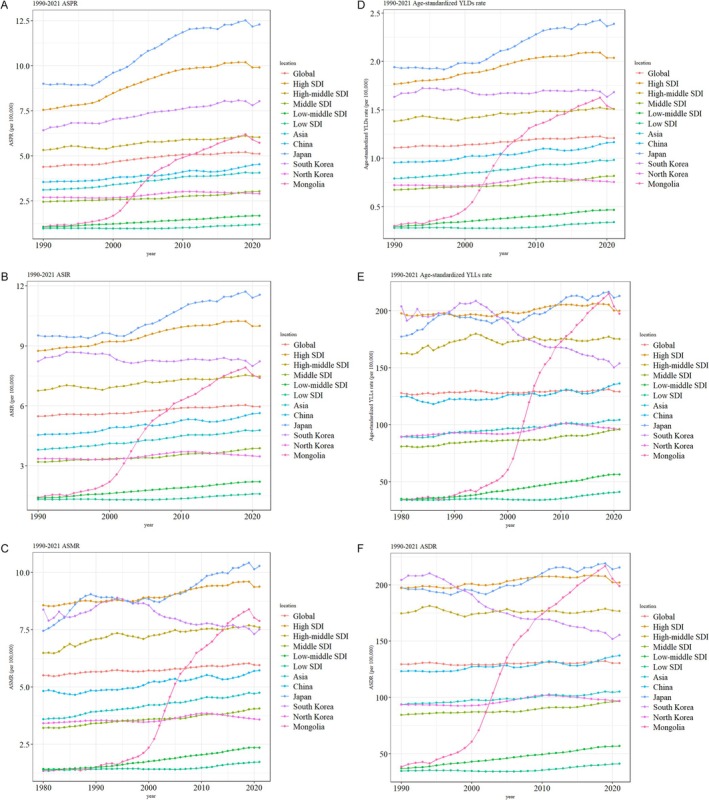
Time trends in the five East Asian countries. (A) Age‐standardized prevalence rate (ASPR), (B) age‐standardized incidence rate (ASIR), (C) age‐standardized mortality rate (ASMR), (D) age‐standardized YLDs rate, (E) age‐standardized YLLs rate, (F) age‐standardized disability‐adjusted life year rate (ASDR).

The ASPR of the five East Asian countries exhibited an overall upward trend from 1990 to 2021 (Figure 3A). For both females and males, the ASPR showed a consistent increase during this period (Figure S1). Among these countries, Japan demonstrated the highest ASPR trends, ranking first. South Korea's ASPR remained above the global level from 1990 to 2021, whereas the ASPRs of China and North Korea were consistently below the global average. Mongolia's ASPR exceeded the global level in 2012 (Figure 3A).

Similarly, the ASIR of the five East Asian countries showed an overall upward trend from 1990 to 2021 (Figure 3B). The ASIR for both females and males followed a similar increasing trajectory during this period (Figure S2). Japan also ranked first in terms of ASIR among the five countries. South Korea's ASIR remained above the global level from 1990 to 2021, whereas those of China and North Korea remained below the global average. Mongolia's ASIR exceeded the global level in 2009 (Figure 3B).

The ASMR of the five East Asian countries exhibited an overall oscillating upward trend from 1990 to 2021 (Figure 3C). For both females and males, the ASMR also displayed a similar oscillating upward pattern over this period (Figure S3). Among these countries, Japan's ASMR trends ranked the highest after exceeding those of South Korea's in 1998. The ASMRs of China and North Korea remained below the global average from 1990 to 2021. In Mongolia, the ASMR exceeded the global level in 2007 and surpassed that of South Korea in 2015 (Figure 3C).

The age‐standardized YLDs rate of the five East Asian countries showed an overall upward trend from 1990 to 2021 (Figure 3D). For both females and males, the YLDs rate demonstrated an oscillating upward trend during this period (Figure S4). Japan had the highest YLDs rate trends among the five countries. South Korea's YLDs remained above the global level from 1990 to 2021, whereas the rates in China and North Korea remained below the global level. In Mongolia, the YLDs exceeded the global average in 2007 (Figure 3D).

The age‐standardized YLLs rate in the five East Asian countries demonstrated an overall upward trend from 1990 to 2021 (Figure 3E). For both females and males in China, Japan, and North Korea, the YLLs exhibited an oscillating upward trend during this period. In contrast, South Korea experienced an oscillating downward trend in YLLs rates for both genders (Figure S5). Japan's YLLs rate ranked the highest among the five countries after surpassing that of South Korea in 1999. North Korea's YLLs rate remained below the global level throughout the period, whereas Mongolia's rate exceeded the global level in 2005 (Figure 3E).

The ASDR in the five East Asian countries displayed an overall oscillating upward trend from 1990 to 2021 (Figure 3F). The ASDR for both females and males in China, Japan, and North Korea showed an oscillating upward trend, whereas South Korea exhibited an oscillating downward trend (Figure S6). Japan's ASDR ranked the highest among the five East Asian countries after exceeding that of South Korea in 1999. North Korea's ASDR remained below the global level from 1990 to 2021, whereas Mongolia's rate exceeded the global level in 2005 (Figure 3F).

### 3.4 Joinpoint Regression Analysis

**FIGURE 4:**
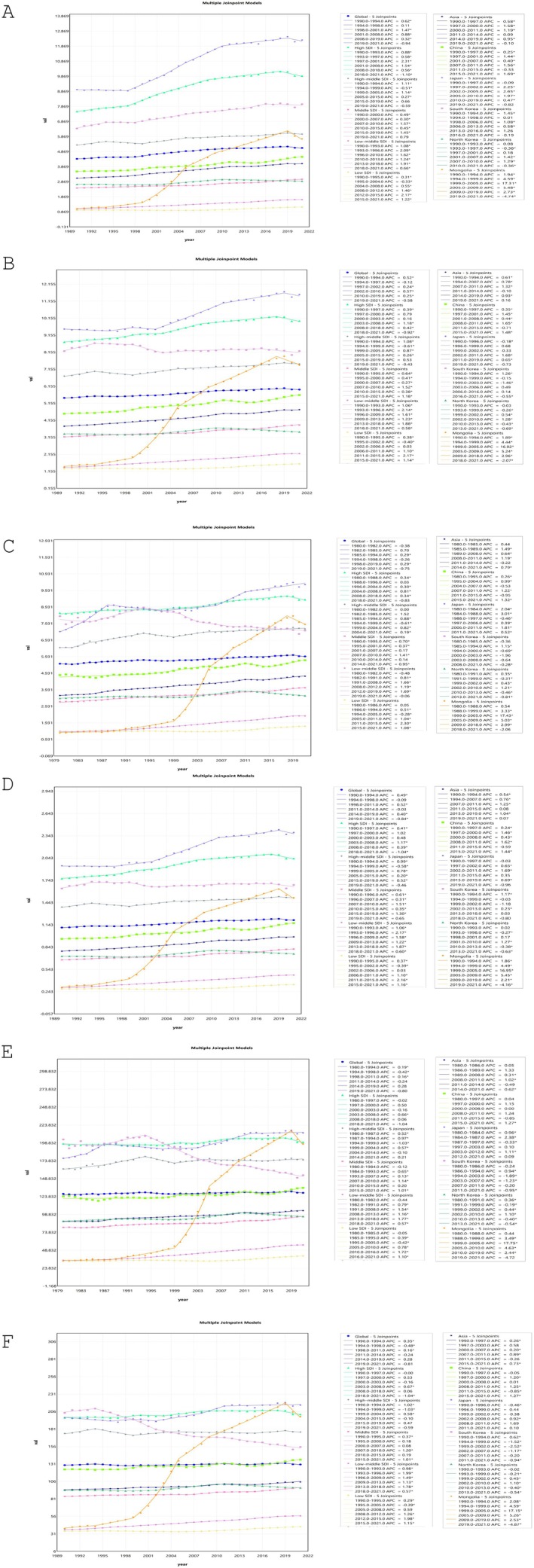
Joinpoint regression analysis of the age‐standardized incidence rate in the five East Asian countries from 1990 to 2021. (A) ASPR, (B) ASIR, (C) ASMR, (D) age‐standardized YLDs rate, (E) age‐standardized YLLs rate, (F) ASDR.

The AAPCs of the ASPR in China [0.80 (95% CI: 0.64, 0.96)], Japan [1.04 (95% CI: 0.97, 1.12)], South Korea [0.67 (95% CI: 0.49, 0.85)], and Mongolia [5.45 (95% CI: 5.13, 5.77)] exceeded the global level [0.49 (95% CI: 0.37, 0.61)]. Conversely, the AAPC of ASPR in North Korea [0.25 (95% CI: 0.18, 0.31)] remained below the global level (Figure 4A and Table S5).

The AAPCs of the ASIR in China [0.72 (95% CI: 0.50, 0.94)], Japan [0.60 (95% CI: 0.42, 0.79)], and Mongolia [5.41 (95% CI: 5.07, 5.75)] exceeded the global level [0.28 (95% CI: 0.17, 0.39)]. Conversely, the AAPC of ASPR in South Korea [−0.05 (95% CI: −0.24, 0.15)] and North Korea [0.11 (95% CI: 0.06, 0.15)] fell below the global level (Figure 4B and Table S5).

The AAPCs of ASMR in China [0.49 (95% CI: 0.29, 0.69)], Japan [0.82 (95% CI: 0.67, 0.96)], and Mongolia [4.42 (95% CI: 4.06, 4.79)] surpassed the global level [0.20 (95% CI: 0.11, 0.28)]. However, the AAPCs of ASMR in South Korea [−0.20 (95% CI: −0.35, −0.04)] and North Korea [0.10 (95% CI: 0.07, 0.14)] were below the global level (Figure 4C and Table S5).

The AAPCs of the age‐standardized YLDs rate in China [0.66 (95% CI: 0.47, 0.86)], Japan [0.66 (95% CI: 0.53, 0.79)], and Mongolia [5.22 (95% CI: 4.90, 5.55)] exceeded the global level [0.28 (95% CI: 0.19, 0.36)]. Conversely, the AAPC of age‐standardized YLDs rate in South Korea [0.04 (95% CI: −0.12, 0.19)] and North Korea [0.14 (95% CI: 0.10, 0.18)] remained below the global level (Figure 4D and Table S5).

The AAPCs of age‐standardized YLLs rate in China [0.29 (95% CI: 0.08, 0.50)], Japan [0.46 (95% CI: 0.30, 0.63)], North Korea [0.17 (95% CI: 0.15, 0.20)], and Mongolia [4.33 (95% CI: 3.88, 4.79)] were higher than the global level [0.04 (95% CI: −0.05, 0.13)]. In contrast, South Korea demonstrated a decline in the AAPC of the YLLs rate [−0.61 (95% CI: −0.76, −0.47)], which was below the global level (Figure 4E and Table S5).

The AAPCs of ASDR in China [0.36 (95% CI: 0.18, 0.54)], Japan [0.31 (95% CI: 0.19, 0.43)], North Korea [0.10 (95% CI: 0.06, 0.15)], and Mongolia [5.34 (95% CI: 5.02, 5.65)] exceeded the global level [0.02 (95% CI: −0.10, 0.15)]. However, South Korea exhibited a significant decline in the AAPC of ASDR [−0.93 (95% CI: −1.14, −0.72)], which was below the global level (Figure 4F and Table S5).

### 3.5 Age‐Period‐Cohort Analysis

**FIGURE 5:**
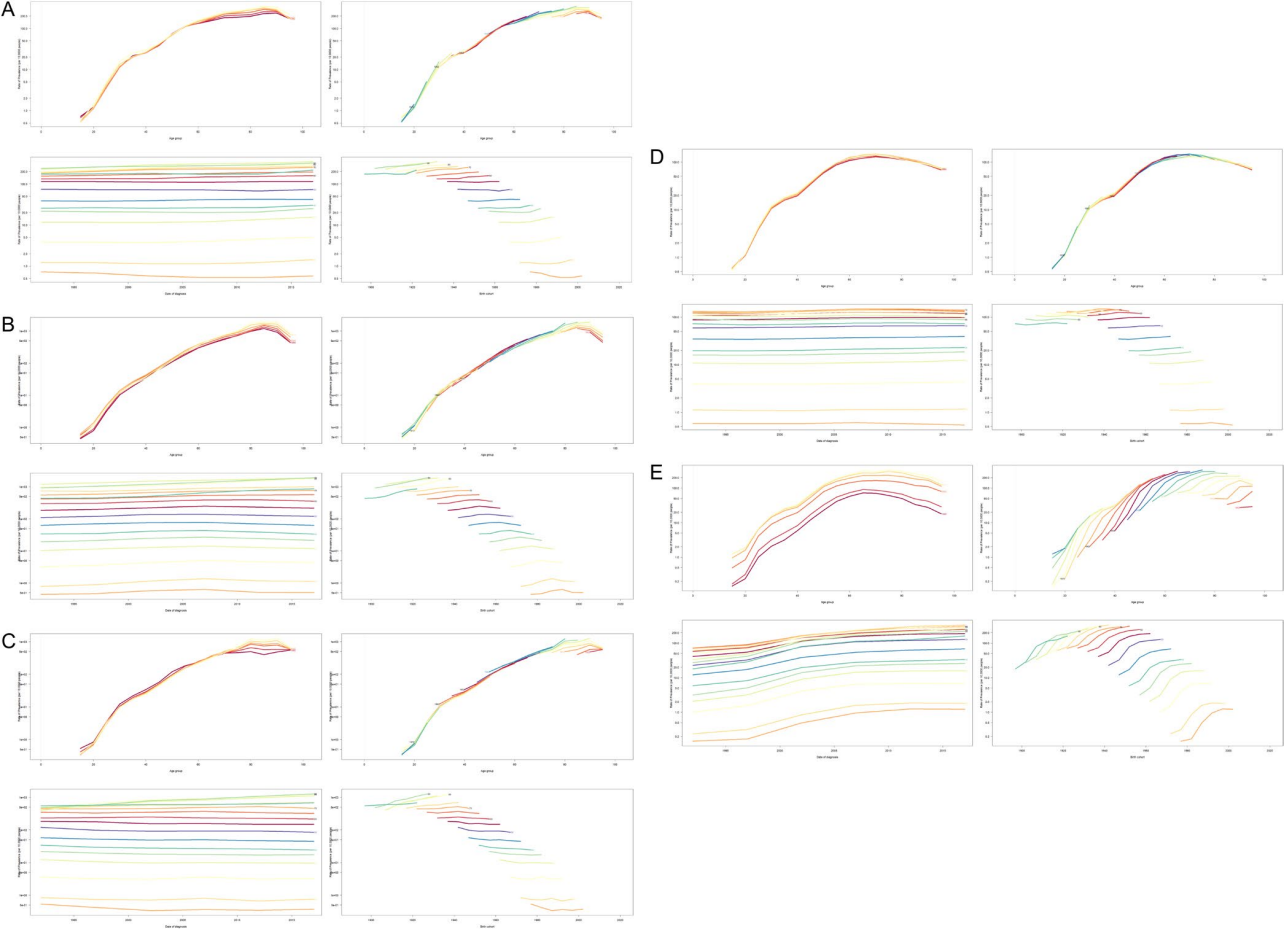
Age‐period‐cohort analysis of prevalence rates. (A) China, (B) Japan, (C) South Korea, (D) North Korea, (E) Mongolia.

The prevalence rate in China increased rapidly between the age of 0 and 90, whereas the prevalence rate exhibited a gradual decline after age 90 (Figure 5A). Similarly, in Japan, the prevalence rate rose rapidly between the age of 0 and 85, while the prevalence rate exhibited a gradual decline after age 85 (Figure 5B). In South Korea, the prevalence rate increased rapidly between the age of 0 and 80, with the prevalence rate declining gradually after age 80 (Figure 5C). North Korea showed a rapid rise in the prevalence rate between the age of 0 and 70, while the prevalence rate displayed a slower declining trend after age 70 (Figure 5D). In Mongolia, the prevalence rate surged significantly from the age 0 to 70, while the prevalence rate displayed a gradual decline after age 70 (Figure 5E).

The incidence rate in China increased rapidly between the age of 0 and 85, and showed a slowly decreasing trend after age 85 (Figure S7A). In Japan, the incidence rate rose sharply from the age 0 to 90 and then declined gradually after age 90 (Figure S7B). The incidence rate in South Korea increased rapidly between the age of 0 and 100 (Figure S7C). In North Korea, the incidence rate surged significantly between the age of 0 to 80 and then exhibited a gradual decline after age 80 (Figure S7D). Similarly, in Mongolia, the incidence rate increased sharply between the age of 0 to 70, with a gradual decline observed after age 70 (Figure S7E).

In China, the death rate rose sharply between the age of 0 and 90, while the death rate exhibited a gradual decline after age 90 (Figure S8A). Similarly, in Japan, the death rate surged significantly from the age 0 to 90, with the death rate gradually declining after age 90 (Figure S8B). In South Korea, the death rate increased rapidly between the age of 0 and 100 (Figure S8C). In North Korea, the death rate surged significantly between the age of 0 and 80, while the death rate displayed a gradual decline after age 80 (Figure S8D). In Mongolia, the death rate increased rapidly between the age 0 and 80, with the death rate showing a slow, decreasing trend after age 80 (Figure S8E).

### 3.6 Decomposition Analysis

**FIGURE 6:**
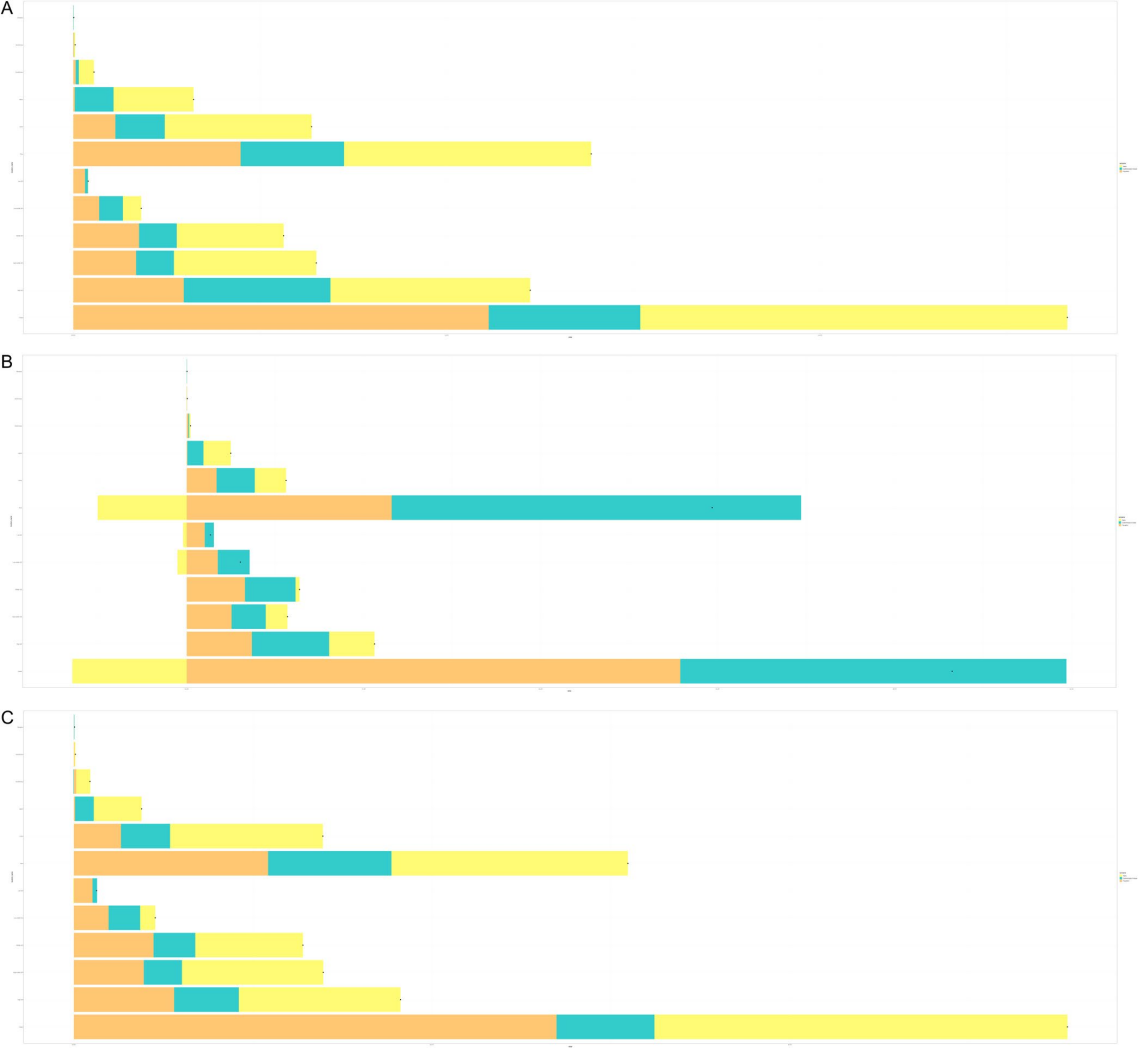
Decomposition analysis. (A) Prevalence, (B) incidence, (C) death.

The prevalence rate in China is influenced by aging (61.58%), population changes (17.68%), and epidemiological shifts (20.75%). In Japan, aging accounts for 66.49% of the prevalence rate, population changes for 1.35%, and epidemiological changes for 32.15%. South Korea's prevalence rate is 73.14% influenced by aging, 11.99% by population, and 14.87% by epidemiological change. In North Korea, aging contributes to 54.62% of the prevalence, population changes to 33.85%, and epidemiological changes to 11.52%. Mongolia's prevalence is predominantly affected by epidemiological changes (61.66%), followed by population (19.90%) and aging (18.44%) (Figure 6A).

The incidence rate in China is 31.48% influenced by aging, 30.10% by population changes, and 38.42% by epidemiological shifts. In Japan, aging contributes to 61.91%, population changes 1.68%, and epidemiological changes 36.41%. South Korea's incidence rate is affected by aging (37.29%) aging, population changes (44.25%), and epidemiological shifts (18.47%). In North Korea, aging accounts for 25.32%, population changes for 65.96%, and epidemiological changes for 8.72%. Mongolia shows a negative impact from aging (−0.91%), with incidence rates primarily influenced by epidemiological changes (78.05%) and population changes (22.86%) (Figure 6B).

The death rate in China is influenced by aging (61.35%), population changes (18.94%), and epidemiological shifts (19.71%). In Japan, aging accounts for 70.53% of the death rate, population changes for 1.78%, and epidemiological changes for 27.69%. South Korea's death rate is predominately affected by aging (86.82%) and population changes (15.48%), with a negative impact from epidemiological changes (−2.30%). In North Korea, aging contributes 56.52%, population changes 43.51%, and epidemiological changes −0.03%. Mongolia's death rate is driven primarily by epidemiological changes (64.93%), followed by population (21.11%) and aging (13.96%) (Figure 6C).

### 3.7 ARIMA Prediction

**FIGURE 7:**
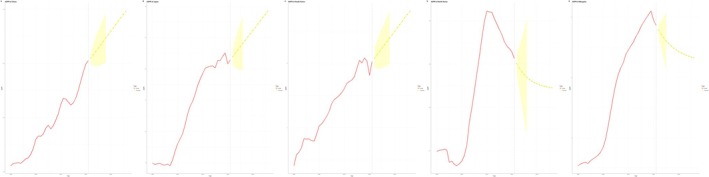
ARIMA prediction of ASPR. (A) China, (B) Japan, (C) South Korea, (D) North Korea, (E) Mongolia.

In China, the ASPR increased from 3.55 in 1990 to 4.53 in 2021, with predictions of 4.80 in 2030 and 4.98 in 2036 (Figure 7A). In Japan, the ASPR rose from 8.99 in 1990 to 12.28 in 2021, with projected increases to 13.23 by 2030 and 13.85 by 2036 (Figure 7B). In South Korea, the ASPR grew from 6.41 in 1990 to 8.02 in 2021, and it is expected to reach 8.49 in 2030 and 8.81 in 2036 (Figure 7C). North Korea's ASPR saw a slight increase from 2.70 in 1990 to 2.91 in 2021, with predictions stabilizing at 2.85 for both 2030 and 2036 (Figure 7D). In Mongolia, the ASPR rose significantly from 1.07 in 1990 to 5.72 in 2021, but is projected to decline to 4.86 in 2030 and 4.65 in 2036 (Figure 7E).

**FIGURE 8:**
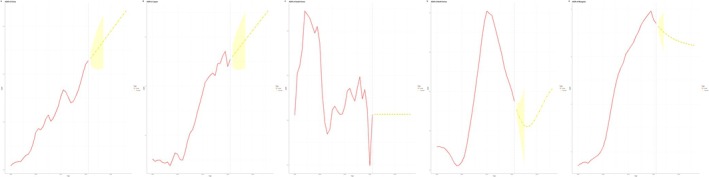
ARIMA prediction of ASIR. (A) China, (B) Japan, (C) South Korea, (D) North Korea, (E) Mongolia.

In China, the ASIR increased from 4.54 in 1990 to 5.64 in 2021, with predicted values of 5.95 in 2030 and 6.16 in 2036 (Figure 8A). In Japan, the ASIR surged from 9.52 in 1990 to 11.55 in 2021, with stable predictions of 12.14 in 2030 and 12.53 in 2036 (Figure 8B). In South Korea, the ASIR changed with values of 8.22 in 1990, 8.23 in 2021, and predicted values of 8.23 for 2030 and 2036 (Figure 8C). In North Korea, the ASIR increased slightly from 3.36 in 1990 to 3.47 in 2021, with projections of 3.44 in 2030 and 3.51 in 2036 (Figure 8D). In Mongolia, the ASIR rose sharply from 1.41 in 1990 to 7.40 in 2021, with expected decreases to 6.65 in 2030 and 6.48 in 2036 (Figure 8E).

**FIGURE 9:**
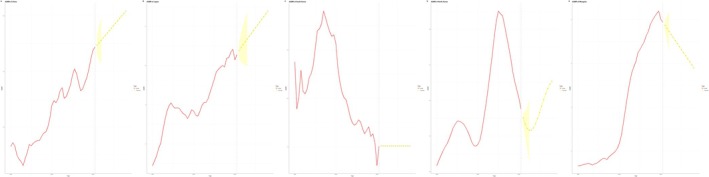
ARIMA prediction of ASMR. (A) China, (B) Japan, (C) South Korea, (D) North Korea, (E) Mongolia.

In China, the ASMR increased from 4.83 in 1990 to 5.72 in 2021, with predictions of 5.91 in 2030 and 6.04 in 2036 (Figure 9A). In Japan, the ASMR rose from 8.96 in 1990 to 10.28 in 2021, and it is projected to reach 10.97 in 2030 and 11.39 in 2036 (Figure 9B). In South Korea, the ASMR decreased from 8.53 in 1990 to 7.51 in 2021, with predictions indicating that it will stabilize at 7.51 for both 2030 and 2036 (Figure 9C). In North Korea, the ASMR slightly increased from 3.54 in 1990 to 3.58 in 2021, with projections of 3.57 in 2030 and 3.65 in 2036 (Figure 9D). In Mongolia, the ASMR surged from 1.50 in 1990 to 7.88 in 2021, but it is expected to decline to 6.63 in 2030 and 5.79 in 2036 (Figure 9E).

### 3.8 Bayesian Age‐Period‐Cohort Prediction

**FIGURE 10:**
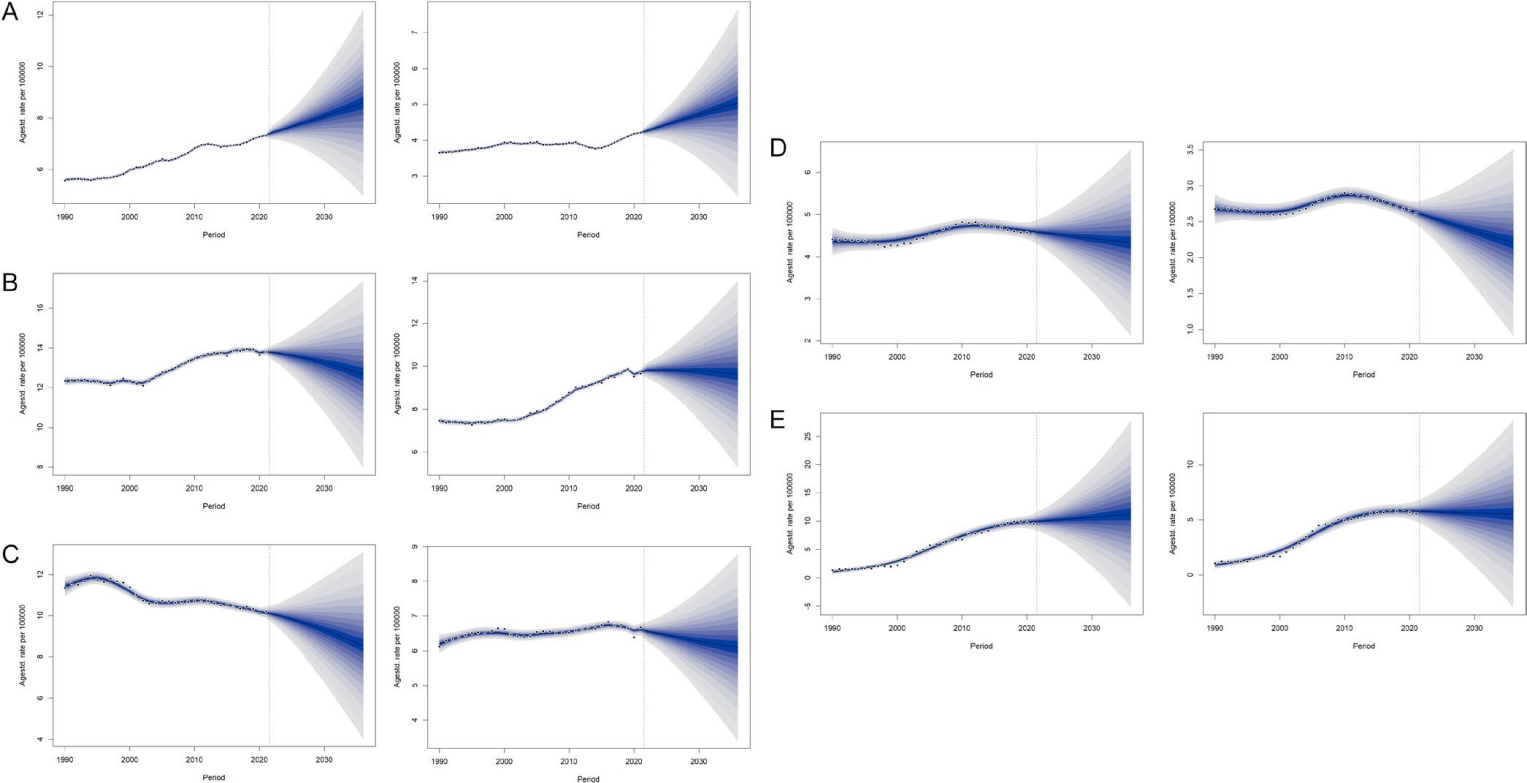
Bayesian age‐period‐cohort prediction of ASIR. (A) China, (B) Japan, (C) South Korea, (D) North Korea, (E) Mongolia.

In China, the ASIR for males increased from 5.60 per 100,000 in 1990 to 7.34 per 100,000 in 2021, with predictions of 8.08 per 100,000 in 2030 and 8.60 per 100,000 in 2036. For females, the ASIR rose from 3.67 per 100,000 in 1990 to 4.22 per 100,000 in 2021, with projections of 4.72 per 100,000 in 2030 and 5.05 per 100,000 in 2036 (Figure 10A).

In Japan, the ASIR for males increased from 12.33 per 100,000 in 1990 to 13.81 per 100,000 in 2021; however, it is expected to decline to 13.25 per 100,000 in 2030 and 12.68 per 100,000 in 2036. For females, the ASIR rose from 7.45 per 100,000 in 1990 to 9.74 per 100,000 in 2021, with predictions of 9.78 per 100,000 in 2030 and 9.64 per 100,000 in 2036 (Figure 10B).

In South Korea, the ASIR for males decreased from 11.41 per 100,000 in 1990 to 10.16 per 100,000 in 2021, and is predicted to further decline to 9.31 per 100,000 in 2030 and 8.56 per 100,000 in 2036. For females, the ASIR rose slightly from 6.20 per 100,000 in 1990 to 6.62 per 100,000 in 2021, but it is expected to decrease to 6.28 per 100,000 in 2030 and 6.10 per 100,000 in 2036 (Figure 10C).

In North Korea, the ASIR for males increased slightly from 4.36 per 100,000 in 1990 to 4.60 per 100,000 in 2021. However, it is predicted to decline to 4.44 per 100,000 in 2030 and 4.33 per 100,000 in 2036. For females, the ASIR decreased from 2.68 per 100,000 in 1990 to 2.63 per 100,000 in 2021, and is expected to further decline to 2.37 per 100,000 in 2030 and 2.21 per 100,000 in 2036 (Figure 10D).

In Mongolia, the ASIR for males rose significantly from 1.12 per 100,000 in 1990 to 9.94 per 100,000 in 2021, and is projected to increase further to 10.69 per 100,000 in 2030 and 11.29 per 100,000 in 2036. For females, the ASIR increased from 0.92 per 100,000 in 1990 to 5.79 per 100,000 in 2021, but is predicted to show a slight decrease, reaching 5.63 per 100,000 in 2030 and 5.54 per 100,000 in 2036 (Figure 10E).

## 4 Discussion

This study provides a comprehensive analysis of the PC burden across five East Asian countries from 1990 to 2021, highlighting significant epidemiological trends and regional disparities. These insights are crucial for informing public health policies and optimizing resource allocation. Our findings indicate that China recorded the highest number of incidence, prevalence, death, YLLs, YLDs, and DALYs among the five countries in both 1990 and 2021. Japan exhibited the highest ASIR, ASMR, ASPR, and age‐standardized YLDs rate in both years. Mongolia experienced the fastest growth in the number and rates of incidence, prevalence, death, YLLs, YLDs, and DALYs from 1990 to 2021. The ASPR, ASIR, ASMR, and age‐standardized YLDs demonstrated an overall upward trend from 1990 to 2021 across the five countries. The AAPCs of ASPR, ASIR, ASMR, age‐standardized YLDs rate, age‐standardized YLLs rate, and ASDR were above the global levels in China, Japan, and Mongolia, with Mongolia recording the highest rates.

In 2021, the age group contributing the highest values of prevalence, incidence, mortality, YLDs, YLLs, and DALYs varied. In China, this group was above 65 years for both genders. In Japan, it was more than 70 years old, whereas in South Korea, it was above 60 years. For North Korea, the highest values were observed in individuals aged ≥ 50 years. In Mongolia, the age group was 55 years and older for both genders. The highest rates of prevalence, incidence, mortality, YLDs, YLLs, and DALYs were consistently observed in individuals aged ≥ 70 years. These findings highlight the significant influence of aging on the burden of PC in East Asia, with incidence surpassing global averages. Mortality and prevalence rates in China, Japan, South Korea, and North Korea, driven by aging also surpassed global levels. All of these factors indicate a significant disease burden on the older adult population.

Gastrointestinal cancers contribute to nearly one‐third of global cancer‐related deaths [18]. Among these, PC has exhibited increasing crude incidence rates from 2000 to 2019 [19]. All indicators of the global burden of PC are on the rise for both males and females, and the ASIR is projected to increase at an alarming rate by 2030 [20, 21]. This trend highlights PC as a growing threat to global health systems, with increasing incidence and mortality observed from 1990 to 2019 [22]. Over the past three decades, the global incidence, mortality, and DALY rates of young‐onset PC have also risen significantly [23]. Certain regions and countries have experienced particularly pronounced increases [23], including Asia, where the ASIR of PC experienced a significant rise from 1990 to 2021 [24].

The burden of early‐onset PC is escalating globally, especially in Asia, with notable high rates observed in Central and Eastern Europe [25]. In 2019, PC attributed to high body mass index (BMI) resulted in 0.7 million DALYs and 31,900 deaths worldwide [26], with the highest burdens observed in high‐income regions such as North America, Western Europe, and East Asia [26]. In China, the incidence, mortality, and DALYs rates of PC have gradually increased over the past three decades, with both absolute numbers and rates expected to continue rising over the next decade [27]. Furthermore, the highest proportion of DALYs attributable to high BMI was reported in the United States and China, each accounting for approximately 15.0% of the burden [28].

Genetic susceptibility to PC in East Asian populations differs from that in other regions [29, 30, 31]. To our knowledge, limited research has focused on the epidemiological patterns of PC across the five East Asian countries. Additionally, ambient temperature and diurnal temperature have been identified as significant factors influencing temperature‐associated mortality, with cardiovascular mortality particularly vulnerable to these environmental changes [32].

We employed ARIMA and BAPC models to predict trends in PC up to 2036. The ASPR, ASIR, and ASMR of China and Japan exhibit a consistent upward trajectory. Similarly, South Korea's ASPR demonstrates a steady increase, whereas its ASIR and ASMR have exhibited an oscillatory decline. In North Korea, the ASPR of North Korea peaked around 2010 and subsequently decreased, while the ASIR declined post‐2010, but is projected to rise again starting in 2027. The ASMR of North Korea increased steadily from 1980 to 1990, declined from 1990 to 1999, rose again from 1999 to 2010, and then fell after peaking around 2010, with another increase anticipated by 2025. In Mongolia, the ASPR, ASIR, and ASMR gradually declined after peaking around 2019.

The growing burden of PC in the five East Asian countries necessitates urgent action in public health policy and prevention strategies. The rising incidence and mortality rates, particularly among older populations, underscore the need to enhance early detection and improve prevention measures. Public health policies should prioritize the establishment of screening programs, especially for high‐risk populations, to facilitate early diagnosis and timely intervention. Additionally, optimizing healthcare resource allocation across regions and ensuring equitable access to care are central goals.

Future strategies must address the increasing disparities in PC burden across countries, with particular focus on the rising case numbers in countries such as China, Japan, and Mongolia. In China, initiatives, such as the Healthy China strategy and coping positively with the aging population strategy, were proposed to enhance awareness of cancer prevention and treatment and promote regular screenings of high‐risk groups, especially older adults. Similarly, in Japan and South Korea, government efforts to improve the quality of life for older adults by strengthening healthcare services and offering targeted educational programs.

Collaboration between governmental health agencies, research institutions, and healthcare providers is essential for implementing effective and regionally tailored health interventions. Furthermore, policies promoting lifestyle changes, such as controlling obesity and promoting healthier diets, should be integrated into national health agendas, given the substantial contribution of high BMI to the PC burden. Additionally, environmental factors, including the health implications of climate change, should be considered when developing future health policies.

Beyond preventive measures, public health strategies should emphasize comprehensive care for patients with PC, incorporating rehabilitation and long‐term care programs to enhance the quality of life for patients and reduce the strain on healthcare systems. In the five East Asian countries, traditional medicine systems, such as traditional Chinese medicine (TCM) and traditional Mongolian medicine [33], have complemented Western medical practices effectively. An integrative approach that combines modern medical treatments with traditional practices may provide synergistic benefits in the prevention and management of PC.

Our study had some limitations. First, while data within each iteration of the GBD are comparable, results across different iterations, even for the same year, are not directly comparable. Discrepancies between iterations may arise from updates in data sources and methodological improvements. Second, the accuracy of the estimated PC burden largely depends on the availability and quality of the underlying data. Although statistical methods can partially mitigate this limitation, estimates in data‐scarce regions, particularly in low socio‐demographic index (SDI) countries, often rely on predictive covariates or global trends adjusted for SDI level and data from a single representative country. These estimates may lack generalizability, warranting cautious interpretation. To improve accuracy, additional high‐quality, population‐based studies are urgently needed in regions with limited data. Third, certain sources of uncertainty, such as the choice of covariates used in the models, are not fully accounted for in our estimations. Additionally, the long‐term impact of the COVID‐19 pandemic could introduce deviations in the prediction models. In future research, we plan to address these limitations by utilizing updated GBD data and other epidemiological databases, further investigating PC trends to enhance the reliability and representativeness of our findings.

In conclusion, the burden of PC in the five East Asian countries has steadily increased over the past three decades, particularly among older adults. Given the complexity of PC pathogenesis, future research should prioritize investigations into etiological factors and the development of effective therapeutic strategies. Public health strategies must focus on enhancing early diagnosis and preventive measures, optimizing the equitable distribution of healthcare resources, and implementing robust environmental health policies. Research on the epidemiology of PC in the populations of the five East Asian countries should be expanded, with a particular emphasis on differentiated management and precise prevention and control strategies for specific regions, genders, and age groups. Additionally, greater attention should be directed toward rehabilitation and long‐term care programs to improve the quality of life for patients with PC and alleviate the burden on society and the healthcare system [34]. Complementary and alternative medicine, when integrated with modern medical treatments, has shown promise in enhancing PC treatment outcomes [35, 36, 37, 38]. By prioritizing early diagnosis, prevention, resource optimization, and integrative approaches, future public health policies can effectively address the increasing burden of PC while improving the quality of care for affected individuals.

## Author Contributions

**Tianhao Guo:** conceptualization (lead), data curation (lead), formal analysis (lead), funding acquisition (supporting), investigation (lead), methodology (lead), project administration (equal), resources (equal), software (lead), supervision (equal), validation (lead), visualization (lead), writing – original draft (lead), writing – review and editing (lead). **Wenjian Zhu:** data curation (equal), formal analysis (equal), investigation (equal), methodology (equal), software (equal), visualization (equal), writing – original draft (equal), writing – review and editing (equal). **Yifan Hui:** data curation (equal), formal analysis (equal), investigation (equal), methodology (equal), supervision (equal), validation (equal), visualization (equal), writing – original draft (equal), writing – review and editing (equal). **Yuhan Wang:** data curation (equal), investigation (equal), methodology (equal), writing – review and editing (equal). **Tingting Zhou:** data curation (equal), formal analysis (equal), investigation (equal), methodology (equal), writing – review and editing (equal). **Weixing Shen:** methodology (equal), project administration (equal), resources (equal), supervision (equal). **Liu Li:** funding acquisition (equal), project administration (equal), supervision (equal). **Yu Yang:** funding acquisition (equal), project administration (equal), resources (equal), supervision (equal). **Haibo Cheng:** conceptualization (equal), funding acquisition (lead), project administration (equal), resources (equal), supervision (equal).

## Ethics Statement

The Affiliated Hospital of Nanjing University of Chinese Medicine Ethics Committee exempted this study from IRB approval due to the use of public domain data. Ethics approval and consent to participate were therefore not required.

## Conflicts of Interest

The authors declare no conflicts of interest.

## Supporting information


Figure S1.

Figure S2.

Figure S3.

Figure S4.

Figure S5.

Figure S6.

Figure S7.

Figure S8.

Table S1.

Table S2.

Table S3.

Table S4.

Table S5.

## Data Availability

Data used for the analyses are publicly available from the Institute of Health Metrics and Evaluation (http://www.healthdata.org/; http://ghdx.healthdata.org/gbd‐results‐tool). The data supporting the findings of this study are from the Global Burden of Disease Study 2021. No restrictions apply to the availability of these data, which were used under Public Use Files (PUF) data. Data are available at https://vizhub.healthdata.org.
