# Supplementary figures and images for: The Burden of Pancreatic Cancer in Five East Asian Countries From 1990 to 2021 and Its Prediction up to 2036: A Systemic Analysis of the Global Burden of Diseases Study 2021
Source: Cancer Med. 2025 Dec 7;14(23):e70656. doi: 10.1002/cam4.70656 (PMC12683073; doi:10.1002/cam4.70656)

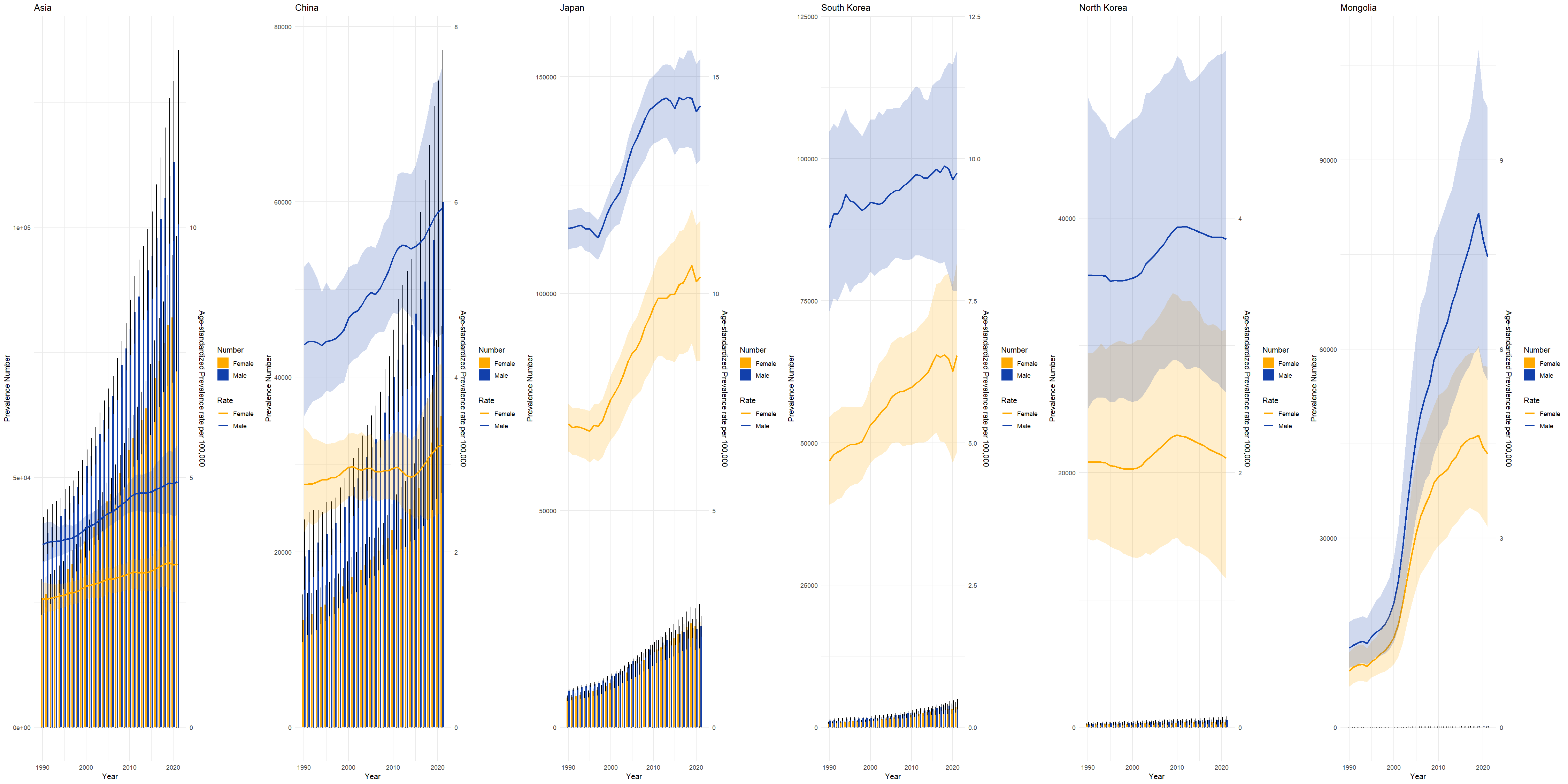

Supplement: Supplementary file 1 — Figure S1. [file CAM4-14-e70656-s004.tiff]

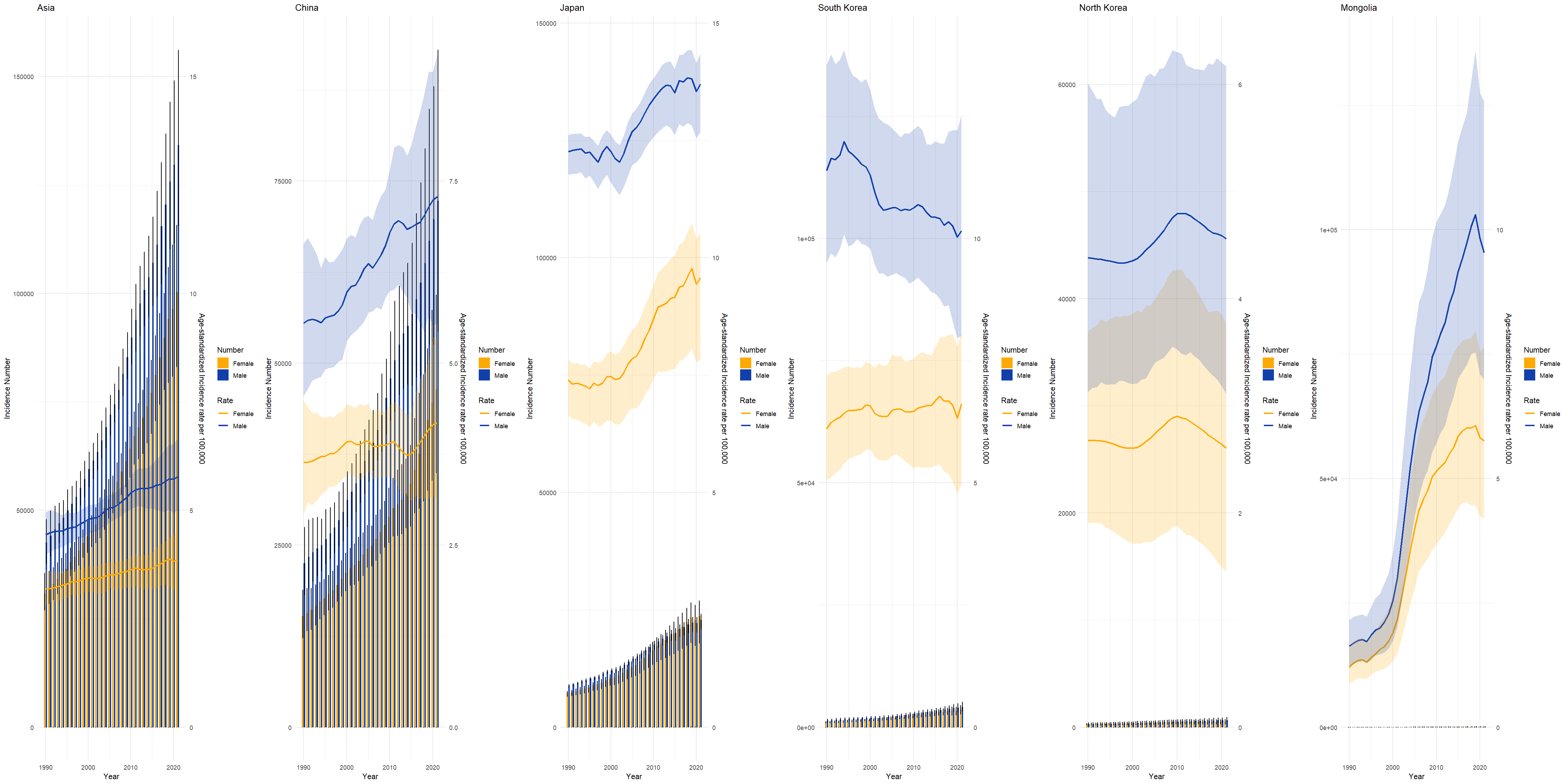

Supplement: Supplementary file 2 — Figure S2. [file CAM4-14-e70656-s002.tiff]

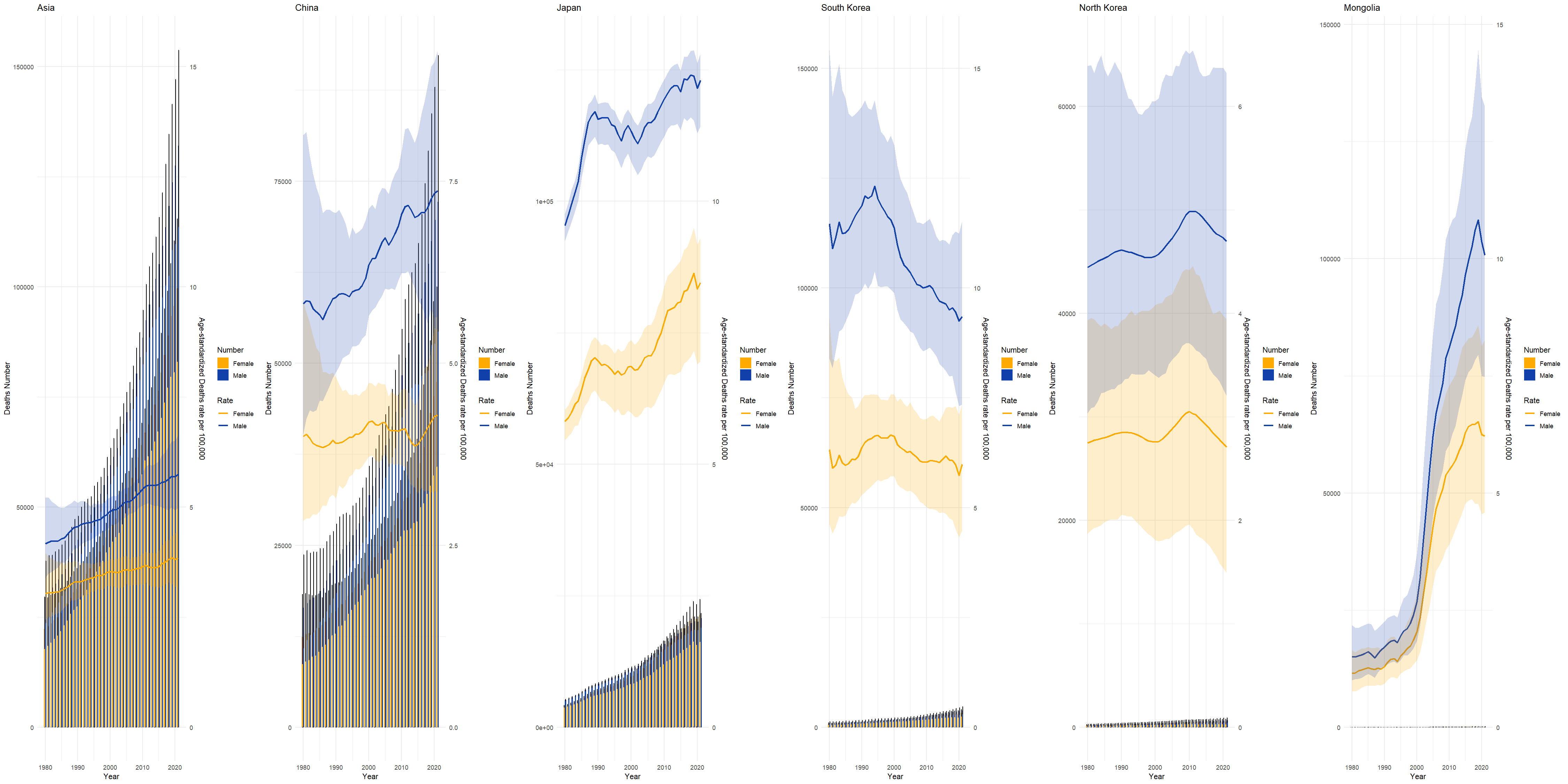

Supplement: Supplementary file 3 — Figure S3. [file CAM4-14-e70656-s010.tiff]

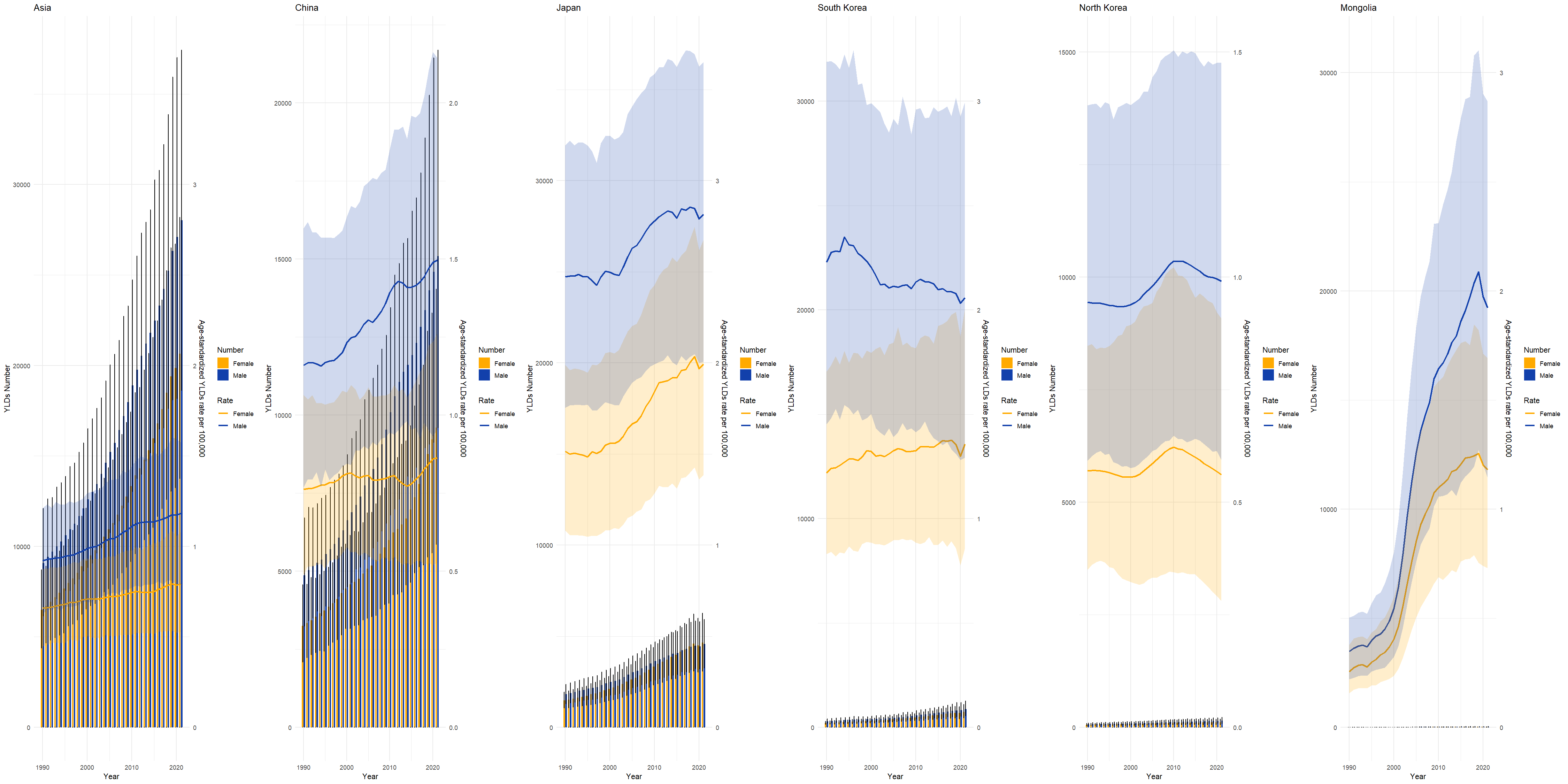

Supplement: Supplementary file 4 — Figure S4. [file CAM4-14-e70656-s007.tiff]

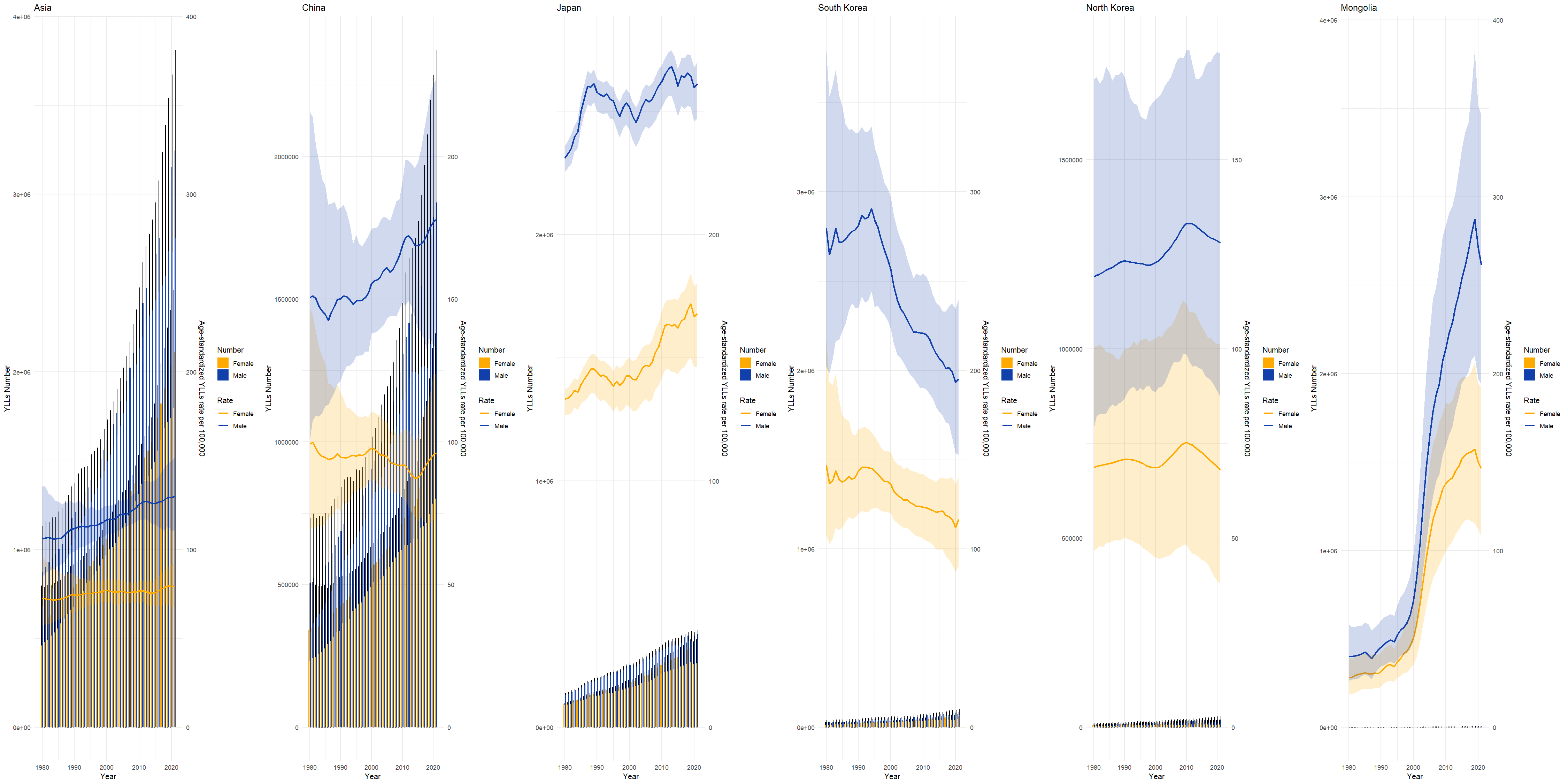

Supplement: Supplementary file 5 — Figure S5. [file CAM4-14-e70656-s001.tiff]

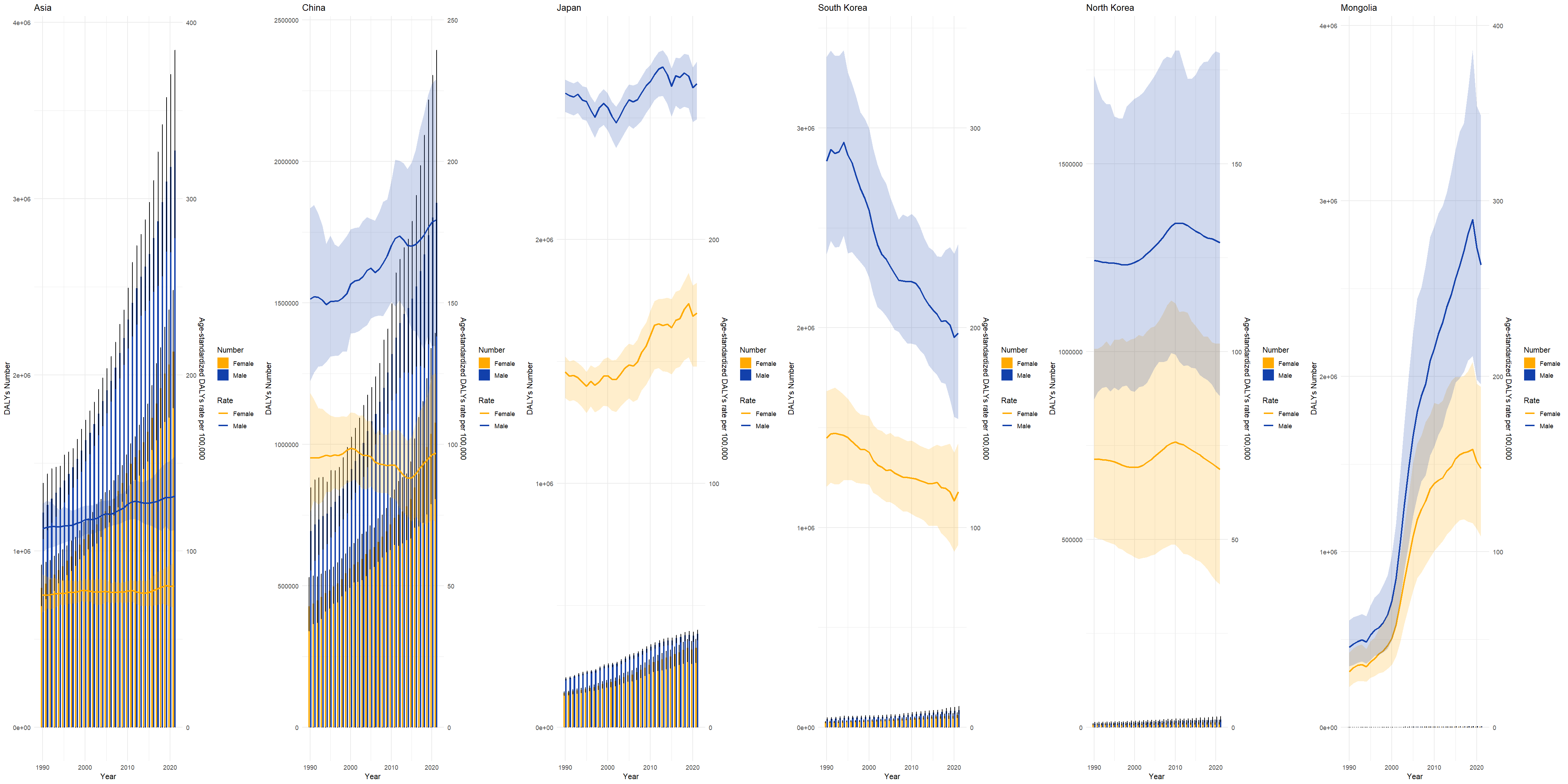

Supplement: Supplementary file 6 — Figure S6. [file CAM4-14-e70656-s005.tiff]

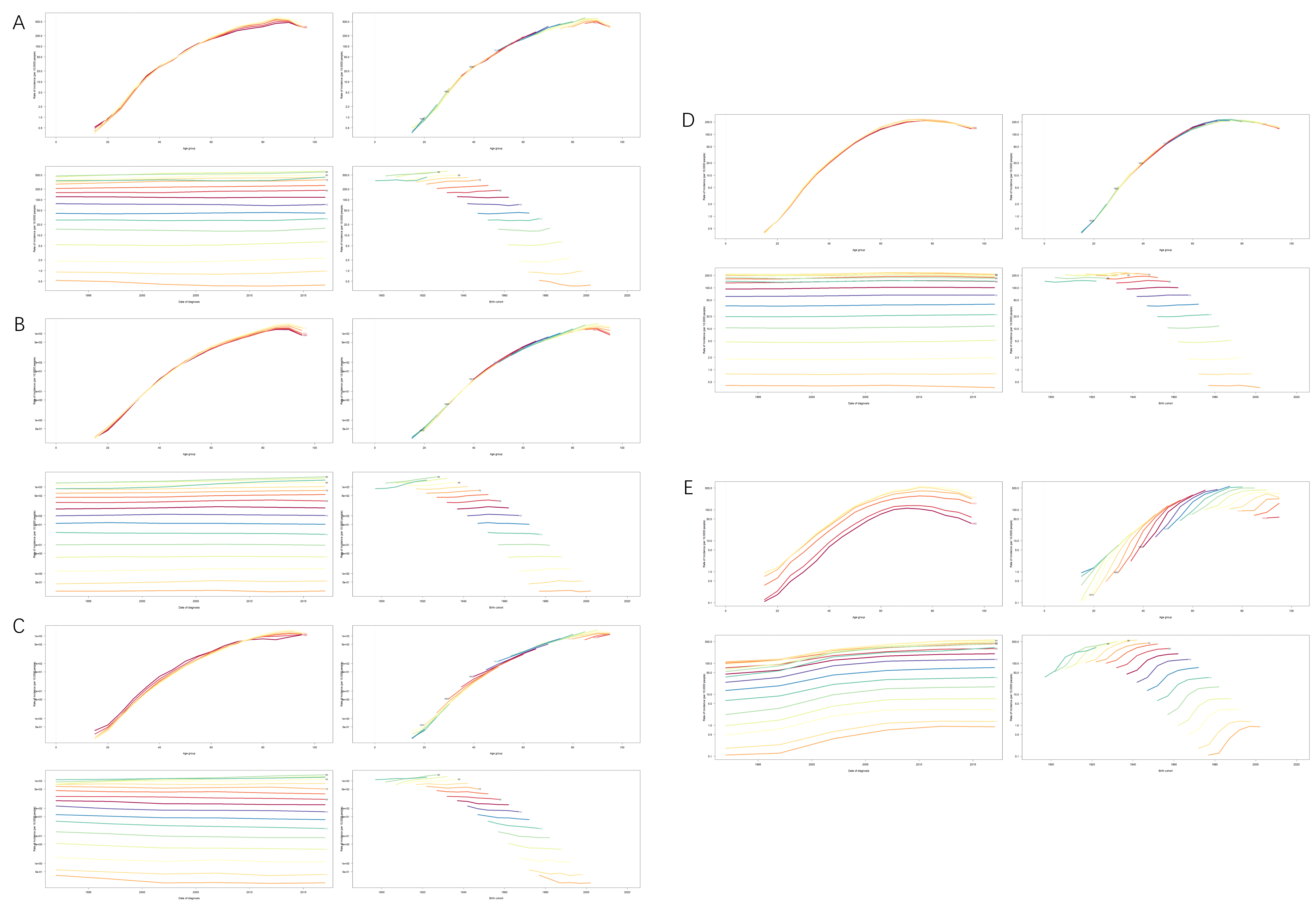

Supplement: Supplementary file 7 — Figure S7. [file CAM4-14-e70656-s012.tif]

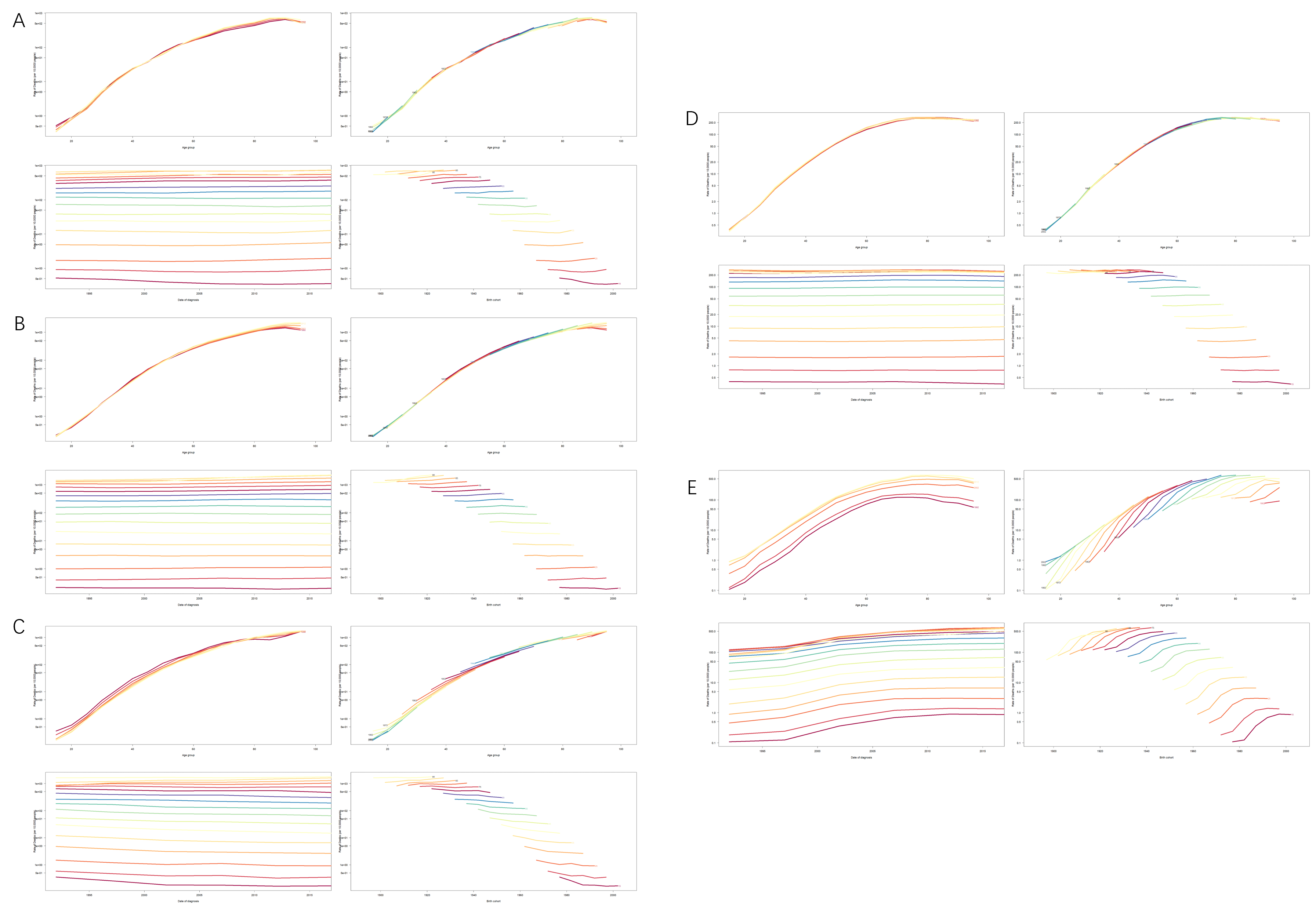

Supplement: Supplementary file 8 — Figure S8. [file CAM4-14-e70656-s008.tif]
