# Supplementary material for: The Burden of Pancreatic Cancer in Five East Asian Countries From 1990 to 2021 and Its Prediction up to 2036: A Systemic Analysis of the Global Burden of Diseases Study 2021
Source: Cancer Med. 2025 Dec 7;14(23):e70656. doi: 10.1002/cam4.70656 (PMC12683073; doi:10.1002/cam4.70656)
Supplement: Supplementary file 9 — Table S1. [file CAM4-14-e70656-s003.docx]

Table S1. Prevalence of Pancreatic Cancer Between 1990 and 2021 at the Global, Regional, and Five East-Asian Countries Levels

| **Location** | **1990 Prevalence cases (95% UI)** |  |  | **1990 Age-standardized rates per 100 000 people (95% UI)** |  |  | **2021 Prevalence cases (95% UI)** |  |  | **2021 Age-standardized rates per 100 000 people (95% UI)** |  |  |
| --- | --- | --- | --- | --- | --- | --- | --- | --- | --- | --- | --- | --- |
|  | **Total** | **Male** | **Female** | **Total** | **Male** | **Female** | **Total** | **Male** | **Female** | **Total** | **Male** | **Female** |
| Global | 172809 (164346,180916) | 94255 (89253,99391) | 78554 (73543,83240) | 4.39 (4.15,4.6) | 5.09 (4.83,5.34) | 3.73 (3.47,3.95) | 439001 (401739,471000) | 238300 (219016,260333) | 200700 (176302,216718) | 5.12 (4.66,5.5) | 5.95 (5.47,6.49) | 4.34 (3.82,4.68) |
| SDI |  | | | | | | | | | | | |
| High SDI | 82919 (79111,85553) | 42565 (41365,43607) | 40354 (37336,42134) | 7.54 (7.2,7.77) | 9.03 (8.76,9.26) | 6.29 (5.87,6.54) | 205270 (184642,218779) | 104475 (97441,110058) | 100795 (85962,109359) | 9.89 (9.05,10.45) | 11.17 (10.47,11.76) | 8.7 (7.67,9.3) |
| High-middle SDI | 53742 (50835,56703) | 30727 (28490,33057) | 23015 (21426,24755) | 5.32 (5.03,5.61) | 6.76 (6.29,7.25) | 4.09 (3.81,4.4) | 118826 (106487,131769) | 67612 (59016,77898) | 51214 (44886,57587) | 6.04 (5.41,6.68) | 7.51 (6.59,8.61) | 4.71 (4.14,5.3) |
| Middle SDI | 26835 (24437,29568) | 15595 (13835,17626) | 11240 (9885,12658) | 2.45 (2.24,2.69) | 2.84 (2.54,3.19) | 2.06 (1.82,2.32) | 83175 (73144,93872) | 48790 (41880,56772) | 34385 (29886,39086) | 3.03 (2.68,3.42) | 3.7 (3.19,4.29) | 2.42 (2.1,2.74) |
| Low-middle SDI | 6767 (5731,7913) | 3928 (3258,4583) | 2839 (2339,3403) | 1.06 (0.9,1.23) | 1.19 (0.99,1.38) | 0.92 (0.76,1.1) | 24972 (23156,27001) | 13817 (12683,14990) | 11156 (10121,12141) | 1.68 (1.56,1.82) | 1.91 (1.75,2.07) | 1.47 (1.33,1.6) |
| Low SDI | 2320 (1801,2782) | 1314 (997,1591) | 1006 (753,1256) | 0.98 (0.76,1.17) | 1.08 (0.82,1.3) | 0.87 (0.65,1.09) | 6332 (5229,7657) | 3379 (2748,4215) | 2953 (2379,3562) | 1.19 (0.99,1.43) | 1.27 (1.05,1.57) | 1.11 (0.9,1.33) |
| Asia | 63256 (57301,70108) | 37435 (33309,41993) | 25821 (22580,29609) | 3.11 (2.83,3.43) | 3.66 (3.29,4.08) | 2.56 (2.26,2.91) | 201962 (177647,227188) | 116870 (100646,135466) | 85092 (71221,98191) | 4.07 (3.56,4.57) | 4.91 (4.26,5.66) | 3.27 (2.72,3.76) |
| China | 31718 (26663,36997) | 19491 (15722,23730) | 12227 (9740,15154) | 3.55 (2.99,4.14) | 4.37 (3.55,5.25) | 2.77 (2.22,3.43) | 95524 (75563,116662) | 59943 (44928,77312) | 35581 (26783,45781) | 4.53 (3.6,5.5) | 5.92 (4.5,7.54) | 3.22 (2.43,4.14) |
| Japan | 15224 (14402,15857) | 8428 (8114,8702) | 6796 (6169,7262) | 8.99 (8.47,9.38) | 11.5 (11.02,11.92) | 6.99 (6.37,7.47) | 47434 (39193,52343) | 23290 (20961,25556) | 24144 (18247,28376) | 12.28 (10.64,13.21) | 14.33 (13.08,15.42) | 10.39 (8.45,11.69) |
| South Korea | 1986 (1689,2285) | 1181 (973,1410) | 805 (673,937) | 6.41 (5.44,7.41) | 8.79 (7.32,10.48) | 4.69 (3.92,5.46) | 7552 (5988,9197) | 4050 (3196,4931) | 3502 (2566,4404) | 8.02 (6.36,9.78) | 9.75 (7.66,11.91) | 6.54 (4.85,8.17) |
| North Korea | 472 (333,659) | 263 (183,373) | 210 (148,301) | 2.7 (1.93,3.71) | 3.55 (2.5,4.95) | 2.08 (1.48,2.94) | 988 (632,1345) | 588 (398,829) | 399 (220,587) | 2.91 (1.86,3.93) | 3.83 (2.63,5.32) | 2.11 (1.17,3.12) |
| Mongolia | 12 (9,15) | 7 (5,9) | 5 (4,7) | 1.07 (0.81,1.4) | 1.26 (0.94,1.67) | 0.89 (0.65,1.19) | 145 (109,191) | 86 (64,113) | 59 (43,77) | 5.72 (4.27,7.58) | 7.46 (5.5,9.84) | 4.34 (3.18,5.72) |
