# Supplementary material for: The Burden of Pancreatic Cancer in Five East Asian Countries From 1990 to 2021 and Its Prediction up to 2036: A Systemic Analysis of the Global Burden of Diseases Study 2021
Source: Cancer Med. 2025 Dec 7;14(23):e70656. doi: 10.1002/cam4.70656 (PMC12683073; doi:10.1002/cam4.70656)
Supplement: Supplementary file 10 — Table S2. [file CAM4-14-e70656-s006.docx]

Table S2. YLDs (Years Lived with Disability) of Pancreatic Cancer Between 1990 and 2021 at the Global, Regional, and Five East-Asian Countries Levels

| **Location** | **1990 YLDs cases (95% UI)** |  |  | **1990 Age-standardized rates per 100 000 people (95% UI)** |  |  | **2021 YLDs cases (95% UI)** |  |  | **2021 Age-standardized rates per 100 000 people (95% UI)** |  |  |
| --- | --- | --- | --- | --- | --- | --- | --- | --- | --- | --- | --- | --- |
|  | **Total** | **Male** | **Female** | **Total** | **Male** | **Female** | **Total** | **Male** | **Female** | **Total** | **Male** | **Female** |
| Global | 42772 (30267,55221) | 22894 (16045,29657) | 19878 (14070,26167) | 1.11 (0.78,1.43) | 1.27 (0.9,1.64) | 0.95 (0.67,1.25) | 103535 (72500,136796) | 56078 (38941,73419) | 47457 (33065,62670) | 1.21 (0.85,1.6) | 1.41 (0.98,1.85) | 1.02 (0.71,1.35) |
| SDI |  | | | | | | | | | | | |
| High SDI | 19568 (13813,25204) | 9895 (7013,12728) | 9672 (6837,12672) | 1.77 (1.25,2.28) | 2.11 (1.5,2.72) | 1.48 (1.05,1.94) | 43132 (30575,56361) | 22146 (15556,28848) | 20986 (14754,27457) | 2.04 (1.44,2.66) | 2.34 (1.64,3.04) | 1.76 (1.24,2.3) |
| High-middle SDI | 13767 (9632,17852) | 7623 (5236,9935) | 6144 (4342,8154) | 1.38 (0.97,1.79) | 1.73 (1.2,2.25) | 1.09 (0.77,1.45) | 29919 (20522,39672) | 16683 (11300,22270) | 13237 (8940,17803) | 1.51 (1.03,2) | 1.86 (1.26,2.48) | 1.2 (0.81,1.61) |
| Middle SDI | 6962 (4762,9299) | 3961 (2659,5275) | 3001 (2028,4004) | 0.67 (0.46,0.89) | 0.77 (0.52,1.02) | 0.58 (0.39,0.76) | 22009 (14905,29227) | 12650 (8322,16882) | 9359 (6244,12801) | 0.82 (0.55,1.08) | 0.98 (0.65,1.31) | 0.66 (0.44,0.91) |
| Low-middle SDI | 1792 (1235,2478) | 1029 (693,1414) | 762 (517,1069) | 0.3 (0.2,0.41) | 0.33 (0.22,0.45) | 0.26 (0.18,0.37) | 6679 (4688,8846) | 3652 (2529,4907) | 3027 (2137,3993) | 0.47 (0.33,0.62) | 0.53 (0.36,0.7) | 0.41 (0.29,0.54) |
| Low SDI | 624 (416,887) | 352 (232,497) | 272 (172,392) | 0.28 (0.19,0.4) | 0.31 (0.2,0.43) | 0.25 (0.16,0.36) | 1688 (1133,2456) | 890 (594,1322) | 798 (542,1149) | 0.34 (0.23,0.49) | 0.36 (0.24,0.53) | 0.32 (0.21,0.46) |
| Asia | 15573 (10580,20666) | 9079 (6143,12096) | 6494 (4365,8710) | 0.79 (0.54,1.04) | 0.92 (0.62,1.21) | 0.66 (0.45,0.88) | 48662 (33067,64965) | 28019 (18619,37430) | 20643 (13751,28180) | 0.98 (0.67,1.31) | 1.18 (0.79,1.58) | 0.79 (0.53,1.08) |
| China | 8111 (5319,11005) | 4863 (3233,6712) | 3248 (2090,4562) | 0.95 (0.63,1.3) | 1.16 (0.77,1.6) | 0.76 (0.49,1.07) | 24697 (15891,34348) | 15092 (9603,21694) | 9604 (5859,14037) | 1.17 (0.75,1.61) | 1.5 (0.95,2.14) | 0.86 (0.53,1.26) |
| Japan | 3292 (2340,4293) | 1813 (1279,2354) | 1480 (1056,1941) | 1.94 (1.38,2.53) | 2.47 (1.75,3.19) | 1.52 (1.08,1.99) | 9203 (6468,12156) | 4565 (3249,5918) | 4639 (3085,6254) | 2.39 (1.68,3.16) | 2.82 (2.01,3.65) | 1.99 (1.39,2.68) |
| South Korea | 487 (326,675) | 283 (187,403) | 204 (140,285) | 1.63 (1.11,2.24) | 2.23 (1.45,3.19) | 1.22 (0.83,1.7) | 1592 (1030,2279) | 860 (547,1250) | 732 (452,1089) | 1.68 (1.09,2.41) | 2.06 (1.29,3) | 1.36 (0.86,2.01) |
| North Korea | 120 (76,176) | 64 (40,96) | 56 (34,83) | 0.72 (0.46,1.04) | 0.94 (0.59,1.38) | 0.57 (0.35,0.85) | 253 (154,385) | 146 (87,219) | 108 (54,175) | 0.75 (0.46,1.13) | 0.99 (0.59,1.48) | 0.56 (0.28,0.91) |
| Mongolia | 3 (2,5) | 2 (1,2) | 1 (1,2) | 0.3 (0.19,0.44) | 0.35 (0.22,0.5) | 0.26 (0.16,0.38) | 36 (23,52) | 21 (13,32) | 15 (9,22) | 1.51 (0.94,2.17) | 1.92 (1.14,2.87) | 1.18 (0.73,1.69) |
