# Supplementary material for: The Burden of Pancreatic Cancer in Five East Asian Countries From 1990 to 2021 and Its Prediction up to 2036: A Systemic Analysis of the Global Burden of Diseases Study 2021
Source: Cancer Med. 2025 Dec 7;14(23):e70656. doi: 10.1002/cam4.70656 (PMC12683073; doi:10.1002/cam4.70656)
Supplement: Supplementary file 11 — Table S3. [file CAM4-14-e70656-s013.docx]

Table S3. YLLs (Years of Life Lost) of Pancreatic Cancer Between 1990 and 2021 at the Global, Regional, and Five East-Asian Countries Levels

| **Location** | **1990 YLLs cases (95% UI)** |  |  | **1990 Age-standardized rates per 100 000 people (95% UI)** |  |  | **2021 YLLs cases (95% UI)** |  |  | **2021 Age-standardized rates per 100 000 people (95% UI)** |  |  |
| --- | --- | --- | --- | --- | --- | --- | --- | --- | --- | --- | --- | --- |
|  | **Total** | **Male** | **Female** | **Total** | **Male** | **Female** | **Total** | **Male** | **Female** | **Total** | **Male** | **Female** |
| Global | 5167707 (4930047,5436060) | 2933440 (2760537,3113744) | 2234266 (2095406,2385586) | 128.22 (121.98,134.81) | 152.93 (144.34,162) | 104.76 (97.9,111.86) | 11213428 (10374402,12053449) | 6406643 (5863500,7042988) | 4806786 (4307827,5202099) | 129.12 (119.47,138.77) | 155.71 (142.55,170.79) | 104.22 (93.47,112.8) |
| SDI |  | | | | | | | | | | | |
| High SDI | 2128953 (2048278,2185764) | 1152172 (1124495,1179313) | 976781 (916598,1012956) | 195.36 (188.02,200.38) | 241.34 (235.19,247.12) | 156.07 (147.43,161.39) | 4010652 (3703848,4232592) | 2193528 (2075387,2308963) | 1817124 (1614490,1943302) | 200 (186.45,210.7) | 237.79 (225.8,249.88) | 164.44 (149.55,174.4) |
| High-middle SDI | 1769657 (1670581,1872326) | 1039332 (960637,1121919) | 730325 (677838,788085) | 173.3 (163.52,183.39) | 224.01 (207.69,241.43) | 129.45 (119.99,139.82) | 3456759 (3092063,3829901) | 2033472 (1772341,2345093) | 1423287 (1256962,1614941) | 175.19 (156.76,193.91) | 222.61 (194.75,255.55) | 131.89 (116.37,149.71) |
| Middle SDI | 937745 (852702,1037028) | 549736 (486292,624309) | 388010 (341058,437910) | 83.93 (76.52,92.31) | 97.82 (87.07,110.31) | 69.96 (61.7,78.82) | 2662701 (2343322,3003605) | 1578791 (1357110,1836118) | 1083910 (943412,1232448) | 95.8 (84.45,107.89) | 117.47 (101.14,136.38) | 75.4 (65.64,85.63) |
| Low-middle SDI | 240115 (202855,281524) | 140285 (116082,163918) | 99830 (82155,119747) | 36.7 (31.1,42.89) | 41.53 (34.52,48.4) | 31.61 (25.96,37.68) | 848589 (785782,917740) | 474317 (434761,515513) | 374272 (339567,409404) | 56.31 (52.24,60.81) | 64.38 (59.12,69.79) | 48.59 (44.03,53.01) |
| Low SDI | 83848 (64999,100894) | 47681 (36270,57695) | 36167 (26928,45168) | 34.54 (26.79,41.39) | 38.29 (29.29,46.3) | 30.65 (22.74,38.31) | 222359 (183076,270533) | 119654 (96833,150445) | 102705 (82319,124740) | 40.87 (33.86,49.35) | 43.99 (36.01,54.49) | 37.77 (30.41,45.61) |
| Asia | 1995017 (1795576,2232374) | 1209818 (1062047,1374447) | 785199 (681191,914280) | 93.08 (83.93,103.69) | 111.72 (98.6,125.91) | 74.24 (64.46,86.02) | 5356260 (4651930,6085781) | 3244108 (2752448,3809942) | 2112153 (1794532,2459970) | 104.28 (91,118.15) | 129.91 (110.9,152.01) | 79.65 (67.49,92.71) |
| China | 1112242 (933929,1297317) | 688375 (551689,840585) | 423866 (337392,526610) | 122.2 (102.85,142.16) | 150.17 (121.46,182.03) | 94.61 (75.54,117.27) | 2905620 (2284585,3543741) | 1838941 (1371658,2372661) | 1066679 (800943,1379845) | 136.06 (107.36,165.26) | 177.86 (133.91,227.18) | 96.03 (72.16,124) |
| Japan | 335396 (320914,344281) | 196848 (191798,200851) | 138548 (128188,144450) | 195.6 (186.65,200.81) | 257.57 (250.04,262.97) | 144.24 (133.73,150.2) | 699862 (617902,746772) | 378441 (353344,393354) | 321421 (260848,356155) | 212.92 (194.11,223.14) | 261.1 (246.97,270.11) | 167.85 (146.22,180.42) |
| South Korea | 64860 (55521,74567) | 39633 (33013,46923) | 25227 (21085,29122) | 202.54 (173.28,233.18) | 281.15 (235.13,333.01) | 143.55 (119.55,166.9) | 144537 (115510,175373) | 84633 (66354,103861) | 59904 (45936,72691) | 153.74 (123.11,186.55) | 195.17 (152.76,239.8) | 116.54 (90.33,141.01) |
| North Korea | 16597 (11655,23164) | 9346 (6500,13209) | 7251 (5098,10407) | 92.9 (66.01,127.89) | 123.33 (86.84,172.02) | 70.77 (50.29,100.02) | 32871 (20960,45482) | 19984 (13409,28501) | 12886 (7104,19092) | 96.07 (61.29,131.55) | 128.03 (87.39,177.95) | 68.09 (37.72,101.3) |
| Mongolia | 420 (320,549) | 237 (180,318) | 183 (133,244) | 38.31 (29.11,50.32) | 45.4 (34.47,60.54) | 31.52 (22.79,42.13) | 5152 (3864,6782) | 3116 (2303,4119) | 2036 (1506,2661) | 197.47 (148.26,261.87) | 261.54 (194.09,346.04) | 146.33 (107.77,192.56) |
