# Supplementary material for: The Burden of Pancreatic Cancer in Five East Asian Countries From 1990 to 2021 and Its Prediction up to 2036: A Systemic Analysis of the Global Burden of Diseases Study 2021
Source: Cancer Med. 2025 Dec 7;14(23):e70656. doi: 10.1002/cam4.70656 (PMC12683073; doi:10.1002/cam4.70656)
Supplement: Supplementary file 12 — Table S4. [file CAM4-14-e70656-s011.docx]

Table S4. DALYs (Disability-Adjusted Life Years) of Pancreatic Cancer Between 1990 and 2021 at the Global, Regional, and Five East-Asian Countries Levels

| **Location** | **1990 DALYs cases (95% UI)** |  |  | **1990 Age-standardized rates per 100 000 people (95% UI)** |  |  | **2021 DALYs cases (95% UI)** |  |  | **2021 Age-standardized rates per 100 000 people (95% UI)** |  |  |
| --- | --- | --- | --- | --- | --- | --- | --- | --- | --- | --- | --- | --- |
|  | **Total** | **Male** | **Female** | **Total** | **Male** | **Female** | **Total** | **Male** | **Female** | **Total** | **Male** | **Female** |
| Global | 5210479 (4967405,5481661) | 2956334 (2782367,3138635) | 2254145 (2113305,2406618) | 129.32 (122.98,135.98) | 154.2 (145.54,163.26) | 105.71 (98.83,112.86) | 11316963 (10464697,12169336) | 6462721 (5913395,7103691) | 4854243 (4346324,5249521) | 130.33 (120.52,140.13) | 157.12 (143.87,172.29) | 105.24 (94.33,113.82) |
| SDI |  | | | | | | | | | | | |
| High SDI | 2148520 (2062653,2203708) | 1162068 (1134076,1188835) | 986453 (925176,1021798) | 197.13 (189.39,202.05) | 243.45 (237.26,249.12) | 157.55 (148.66,162.74) | 4053784 (3742275,4283639) | 2215674 (2098045,2329579) | 1838110 (1634566,1964802) | 202.04 (188.27,212.61) | 240.12 (227.79,252.35) | 166.2 (151.3,176.2) |
| High-middle SDI | 1783424 (1682492,1890335) | 1046955 (967686,1131280) | 736469 (683516,794423) | 174.68 (164.79,185.04) | 225.74 (209.27,243.59) | 130.55 (121,140.94) | 3486679 (3118893,3861181) | 2050155 (1787608,2365087) | 1436524 (1266667,1629195) | 176.7 (158.07,195.43) | 224.47 (196.3,257.75) | 133.09 (117.16,150.95) |
| Middle SDI | 944708 (858506,1044034) | 553697 (489592,629149) | 391011 (343744,441173) | 84.6 (77.1,92.93) | 98.59 (87.75,111.19) | 70.53 (62.27,79.45) | 2684711 (2362750,3027672) | 1591441 (1367129,1850415) | 1093270 (950621,1245134) | 96.61 (85.18,108.88) | 118.45 (101.99,137.45) | 76.07 (66.13,86.52) |
| Low-middle SDI | 241906 (204322,283539) | 141314 (116958,165123) | 100592 (82778,120953) | 36.99 (31.34,43.21) | 41.86 (34.8,48.79) | 31.87 (26.23,38.05) | 855268 (792108,926183) | 477969 (438202,518899) | 377299 (342485,412434) | 56.78 (52.66,61.42) | 64.9 (59.62,70.34) | 49 (44.42,53.41) |
| Low SDI | 84473 (65503,101441) | 48033 (36566,58114) | 36440 (27083,45546) | 34.82 (27.01,41.7) | 38.59 (29.56,46.71) | 30.9 (22.89,38.61) | 224047 (184707,272778) | 120545 (97570,151557) | 103502 (83025,125748) | 41.21 (34.19,49.79) | 44.35 (36.31,55.02) | 38.09 (30.69,45.98) |
| Asia | 2010590 (1809748,2243943) | 1218897 (1070131,1384791) | 791693 (688274,921934) | 93.87 (84.72,104.39) | 112.64 (99.4,126.87) | 74.9 (65.01,86.78) | 5404922 (4693887,6135548) | 3272126 (2775721,3843304) | 2132796 (1811250,2479646) | 105.26 (91.81,119.1) | 131.09 (111.96,153.41) | 80.44 (68.1,93.47) |
| China | 1120353 (941076,1306509) | 693239 (555482,845950) | 427114 (339532,530296) | 123.16 (103.69,143.27) | 151.33 (122.42,183.53) | 95.37 (76.11,118.14) | 2930317 (2301049,3575079) | 1854033 (1382248,2393909) | 1076284 (806636,1392992) | 137.23 (108.15,166.74) | 179.36 (134.98,229.1) | 96.89 (72.71,125.18) |
| Japan | 338688 (323968,347694) | 198660 (193395,202933) | 140028 (129569,146149) | 197.54 (188.57,202.98) | 260.05 (252.35,265.6) | 145.76 (135.1,151.98) | 709065 (625627,755944) | 383006 (357658,398509) | 326060 (264552,361333) | 215.31 (196.04,225.71) | 263.92 (249.43,273.03) | 169.84 (147.93,182.35) |
| South Korea | 65347 (55925,75064) | 39916 (33181,47246) | 25431 (21252,29330) | 204.18 (174.62,234.83) | 283.38 (236.91,335.6) | 144.77 (120.58,168.27) | 146129 (116596,177361) | 85494 (67016,104928) | 60635 (46524,73381) | 155.42 (124.26,188.67) | 197.23 (154.28,242.11) | 117.9 (91.48,142.35) |
| North Korea | 16717 (11730,23329) | 9410 (6543,13299) | 7307 (5133,10481) | 93.62 (66.55,128.98) | 124.28 (87.38,173.38) | 71.34 (50.64,100.7) | 33124 (21164,45864) | 20130 (13500,28730) | 12994 (7162,19241) | 96.83 (61.86,132.78) | 129.02 (88.1,179.42) | 68.65 (38.02,102.09) |
| Mongolia | 424 (322,554) | 239 (182,320) | 185 (134,246) | 38.61 (29.32,50.7) | 45.75 (34.73,60.99) | 31.78 (22.96,42.46) | 5189 (3889,6827) | 3137 (2319,4147) | 2051 (1516,2683) | 198.98 (149.16,263.83) | 263.47 (195.41,348.52) | 147.51 (108.56,194.09) |
