# Supplementary material for: The Burden of Pancreatic Cancer in Five East Asian Countries From 1990 to 2021 and Its Prediction up to 2036: A Systemic Analysis of the Global Burden of Diseases Study 2021
Source: Cancer Med. 2025 Dec 7;14(23):e70656. doi: 10.1002/cam4.70656 (PMC12683073; doi:10.1002/cam4.70656)
Supplement: Supplementary file 13 — Table S5. [file CAM4-14-e70656-s009.docx]

Table S5. AAPC of Pancreatic Cancer From 1990 to 2021 at the Global, Regional and Five East-Asian Countries Levels

| **Location** | **ASPR** **(95% CI)** | **ASIR** **(95% CI)** | **ASMR** **(95% CI)** | **Age-standardized YLDs rate** **(95% CI)** | **Age-standardized YLLs rate** **(95% CI)** | **ASDR (95% CI)** |
| --- | --- | --- | --- | --- | --- | --- |
| Global | 0.49 (0.37, 0.61) | 0.28 (0.17, 0.39) | 0.20 (0.11, 0.28) | 0.28 (0.19, 0.36) | 0.04 (-0.05, 0.13) | 0.02 (-0.10, 0.15) |
| SDI |  |  |  |  |  |  |
| High SDI | 0.87 (0.78, 0.96) | 0.43 (0.32, 0.53) | 0.21 (0.11, 0.31) | 0.45 (0.36, 0.53) | 0.03 (-0.09, 0.16) | 0.06 (-0.08, 0.21) |
| High-middle SDI | 0.45 (0.36, 0.53) | 0.35 (0.28, 0.43) | 0.42 (0.34, 0.50) | 0.31 (0.22, 0.39) | 0.21 (0.12, 0.30) | 0.04 (-0.08, 0.16) |
| Middle SDI | 0.71 (0.62, 0.79) | 0.67 (0.56, 0.77) | 0.61 (0.52, 0.71) | 0.64 (0.53, 0.75) | 0.47 (0.39, 0.54) | 0.45 (0.36, 0.53) |
| Low-middle SDI | 1.55 (1.48, 1.63) | 1.52 (1.45, 1.60) | 1.28 (1.20, 1.35) | 1.49 (1.37, 1.62) | 1.22 (1.16, 1.28) | 1.40 (1.27, 1.53) |
| Low SDI | 0.66 (0.59, 0.73) | 0.65 (0.57, 0.73) | 0.56 (0.50, 0.63) | 0.65 (0.56, 0.74) | 0.47 (0.42, 0.51) | 0.55 (0.44, 0.67) |
| Asia | 0.86 (0.72, 1.01) | 0.77 (0.63, 0.91) | 0.70 (0.55, 0.85) | 0.71 (0.59, 0.83) | 0.39 (0.23, 0.56) | 0.38 (0.25, 0.51) |
| China | 0.80 (0.64, 0.96) | 0.72 (0.50, 0.94) | 0.49 (0.29, 0.69) | 0.66 (0.47, 0.86) | 0.29 (0.08, 0.50) | 0.36 (0.18, 0.54) |
| Japan | 1.04 (0.97, 1.12) | 0.60 (0.42, 0.79) | 0.82 (0.67, 0.96) | 0.66 (0.53, 0.79) | 0.46 (0.30, 0.63) | 0.31 (0.19, 0.43) |
| South Korea | 0.67 (0.49, 0.85) | -0.05 (-0.24, 0.15) | -0.20 (-0.35, -0.04) | 0.04 (-0.12, 0.19) | -0.61 (-0.76, -0.47) | -0.93 (-1.14, -0.72) |
| North Korea | 0.25 (0.18, 0.31) | 0.11 (0.06, 0.15) | 0.10 (0.07, 0.14) | 0.14 (0.10, 0.18) | 0.17 (0.15, 0.20) | 0.10 (0.06, 0.15) |
| Mongolia | 5.45 (5.13, 5.77) | 5.41 (5.07, 5.75) | 4.42 (4.06, 4.79) | 5.22 (4.90, 5.55) | 4.33 (3.88, 4.79) | 5.34 (5.02, 5.65) |
